# Hybrid CFD and machine learning analysis of CO_2_ enhanced oil recovery in naturally fractured reservoirs

**DOI:** 10.1038/s41598-025-34786-7

**Published:** 2026-01-07

**Authors:** Taimoor Asim, Khaliq Ur Rahman, Haval Kukha Hawez, Rakesh Mishra

**Affiliations:** 1 School of Computing, Engineering and Technology, Robert Gordon University, Aberdeen, UK; 2 Department of Chemical Engineering, Faculty of Engineering, Koya University, Kurdistan Region – F.R, Koya, KOY45 Iraq; 3 School of Computing and Engineering, University of Huddersfield, Huddersfield, HD1 3DH UK

**Keywords:** CO_2_ geosequestration, Enhanced oil recovery (EOR), Naturally fractured reservoirs, Computational fluid dynamics (CFD), Machine learning (ML), Discrete fracture model (DFM), Gaussian process regression (GPR), Extreme gradient boosting (XGBoost), Energy science and technology, Engineering, Mathematics and computing

## Abstract

CO_2_ based Enhanced Oil Recovery (EOR) in unconventional reservoirs is an emerging technology. Scientific research efforts are directed towards understanding the propagation of CO_2_ front due to the complex interplay between CO_2_ injection and saturation, and reservoir’s constitutive relationships. Conventional methods for characterising CO_2_-EOR rely on high-fidelity numerical solutions that often result in over or under prediction of CO_2_ geosequestration. In this study, we develop a novel hybrid Computational Fluid Dynamics (CFD) and Machine Learning (ML) framework that allows for rapid CO_2_ geosequestration prediction and its optimal injection. Very low or high injection rates have been shown to result in low sweep efficiency or excessive entry pressure, while an intermediate injection rate offers the best balance between the two. CFD data-driven Gaussian Process Regression (GPR) and Extreme Gradient Boosting (XGBoost) models have been developed, trained and tested for predicting CO_2_ saturation in the reservoir. Comparative analysis indicates that GPR outperforms XGBoost in terms of its predictive performance and robustness. Through the analysis of layer-resolved CO_2_ front displacement and development of data-driven surrogate models, this study contributes a novel framework for CO_2_-EOR predictive modelling and optimising injection strategies in naturally fractured reservoirs.

## Introduction

United Nations sustainable development goal 13 on climate action states that there is an urgent need to reduce atmospheric CO_2_ in order to combat the effects of global warming^1^. Although average per capita CO_2_e emissions is 6.2 tonnes (as of 2019), the country-wise emissions chart provided by the World Bank clearly shows that this value is much higher (exceeding 25 tonnes) in oil rich countries like the Middle East, Russia, USA, Canada, Libya and Australia^2^. A relatively recent technology that has surfaced to prominence, especially in the context of unconventional reservoirs like fractured rocks and has the potential of (passively) decarbonizing the oil extraction process is CO_2_ based Enhanced Oil Recovery (EOR)^3^. Simply put, in this technology CO_2_ is injected into oil containing formations. CO_2_ pushes the oil out while it stays there and remains stored for long periods of time (several hundred years). This long-term storage of CO_2_ in geological formations is commonly known as CO_2_ geosequestration^4^. In favourable conditions, CO_2_ reacts with the rock material, converting it into a mineral^5^. CO_2_ geosequestration is undergoing field trials at different locations globally. Initial success of these projects indicates the viability of large-scale CO_2_ storage in natural formations^6,7^. However, there are still many challenges which are yet to be resolved. In terms of the science behind this technology, one of the key questions is how much CO_2_ can be stored in geological formations with the required margin of safety. A massive scientific effort is currently undergoing globally to address this question. The key reason behind this is the wide variety of geological formations around the world, for instance, unfractured porous rocks, naturally fractured rocks, tight formations, caprocks, etc. with each having its own unique properties. If the sole purpose is CO_2_ storage, unfractured or lightly fractured saline aquifers are most suitable, however, if oil extraction is also required then naturally fractured rocks are favoured, and thus, this study focuses on CO_2_-EOR in naturally fractured reservoirs.

Algharaib and Alajmi^8^ developed robust analytical tools to evaluate both the oil recovery factor and the CO₂ storage capacity in CO_2_-EOR projects. These evaluations are significant for assessing both the economic viability and environmental impact of such projects. They used a CO_2_ Miscible Flood Predictive Model (MFPM) to generate a large dataset (353 scenarios) covering a wide range of realistic reservoir and operational conditions. The developed model is validated against both the MFPM outputs and existing published models, finding excellent agreement and general applicability. Furthermore, the study supports the dual role of CO_2_-EOR in enhancing oil recovery while facilitating the long-term geological storage of CO_2_, thereby contributing to climate mitigation efforts. Chen et al.^9^ investigated the effectiveness of Injection-Production Coupling (IPC) technique in improving CO_2_-EOR performance in heterogeneous oil reservoirs. They explored the impact of different IPC strategies, synchronous and asynchronous cycles, on oil recovery, CO_2_ utilization, and sweep efficiency in reservoirs with varying permeability structures. The research combines microfluidic visualization experiments, core flooding tests, and numerical simulations to understand the complex fluid dynamics during CO_2_ injection. Asynchronous IPC (staggered injection and production) significantly outperformed synchronous injection, enhancing CO_2_ sweep efficiency and oil displacement, particularly in low-permeability zones. The IPC mechanism delays gas channelling and improves vertical and areal sweep, which are typically problematic in layered or fractured formations. High CO_2_ mobility still leads to early breakthrough in high-permeability zones, but IPC mitigates this by better managing pressure gradients and flow paths. The simulation results closely match the laboratory observations, confirming the effectiveness of the IPC in diverse geological settings. Zhang et al.^10^ investigated the effects of varying CO_2_ injection rates on oil recovery efficiency and gas channelling behaviour in fractured, low-permeability reservoirs. The study utilizes numerical simulation models to evaluate the effect of CO_2_ injection rates on the interaction between fractures and the matrix in reservoir systems with low permeability. The research focuses on understanding gas channelling dynamics and optimizing injection strategies to enhance oil displacement and CO_2_ geosequestration. The results highlight that high injection rates reduce early CO_2_ breakthrough and suppress gas channelling by increasing pressure gradients, which enhances sweep efficiency in the matrix. Moreover, fracture-matrix mass transfer improves at higher injection rates, leading to better utilisation of the matrix’s oil potential. Furthermore, at low injection rates, CO_2_ preferentially flows through fractures, bypassing the matrix and resulting in poor sweep efficiency and early gas breakthrough. An optimal injection rate exists that balances EOR and CO_2_ storage while minimizing the risks of gas channelling.

Dehury and Sangwai^11^ investigate how fluid properties and surface interactions influence CO_2_ storage mechanisms in porous geological formations, particularly in saline aquifers. Microfluidic experiments mimicked porous rocks, varying salinity, wettability and IFT to study CO_2_ flow dynamics. Computational Fluid Dynamics (CFD) based simulations were utilized to model immiscible CO_2_-brine flow under reservoir conditions, analysing fingering, breakthrough time, saturation and interfacial area using the Volume of Fluid (VOF) method. The results indicate that high brine salinity enhances CO_2_ saturation and continuity, favouring structural and mineral trapping, but reduces residual trapping. Lowering interfacial tension using surfactants increases CO_2_-brine contact, thereby promoting solubility and capillary trapping. Water-wet conditions Favor residual trapping, while CO_2_-wet conditions support structural trapping. High reservoir temperature and pressure promote the formation of supercritical CO_2_, enhancing mobility, saturation and breakthrough time, while mitigating viscous fingering instability, resulting in more efficient storage. Gao et al.^12^ investigated how variations in Capillary Pressure (P_c_) across layered heterogeneous formations affect CO_2_ trapping after injection. The study employed numerical simulations to investigate CO_2_ trapping in a 2D heterogeneous saline aquifer using MATLAB’s Reservoir Simulation Toolbox (MRST). CO_2_ injection and brine backflow are modelled, incorporating variations in permeability, capillary pressure and layer slopes. Key mechanisms include capillary heterogeneity-induced trapping, where CO_2_ accumulates beneath higher-entry-pressure layers, residual trapping, driven by brine displacing CO_2_ during post-injection, and structural trapping where sloped layering and permeability contrasts enhance CO_2_ pooling and immobilization through gravity-driven migration and capillary barriers. The results show that P_c_ strongly affects post-injection CO_2_ trapping. Sloped layers and anisotropy promote lateral migration, while residual trapping improves as CO_2_ is halted at capillary barriers displaced by brine during imbibition.

Applying CO_2_-EOR methods to fractured reservoirs raises several difficulties that significantly complicate the process^13,14^. One of the main challenges is the ultra-low permeability of the rock matrix in these formations^15^. Unless a network of fractures is present to enable CO_2_ propagation through them, this very low permeability restricts the flow of CO_2_ through the reservoir. Thus, making it challenging for CO_2_ to enter the matrix and push the trapped oil efficiently^16^. There is considerable variation in the spacing, opening, direction, and connectivity of the fractures inside naturally fractured reservoirs. Uneven sweep efficiency and localized CO_2_ breakthroughs could occur because of the distinct fluid flow routes that this diversity creates, which would make the oil recovery process less effective^17^. Transport processes in fractured reservoirs also occur across several sizes, from nanometer-scale pores in the rock matrix to larger-scale fracture networks. Advanced modelling methods that precisely depict interactions between the matrix and fractures, including the dual-porosity or dual-permeability flow behaviour, are required for this multi-scale phenomenon^18,19^. An exact representation of such complex flow dynamics will assist in predicting how CO_2_ will propagate through the reservoir and interact with the oil. Furthermore, fractures in geological formations are not constant and often respond dynamically to changes in reservoir conditions, particularly pressure and stress, during CO_2_ injection^20^. Designing efficient injection plans, maximizing excellent oil recovery, and assessing the viability and safety of CO_2_ geosequestration depends on precise and sophisticated CFD modelling of CO_2_-EOR in fractured reservoirs^21^. Moreover, the significant disparity between matrix and fracture permeability and limited fracture volumes makes the prediction of fluid flow in naturally fractured reservoirs difficult. Consequently, multiple methodologies have been proposed to represent non-functional requirements.

Dual Continuum models are the predominant modelling approach for non-fuel resources, although Discrete Fracture Models (DFMs) are also gaining interest amongst the researchers to study CO_2_-EOR. The dual-porosity model for single-phase systems was the first dual-continuum model developed^22^. In this model, an orthorhombic continuum of fractures separates straight prisms of the matrix (the sugar-cube model). In dual-porosity model, the reservoir is split into two parts i.e. the matrix and the fracture. Hence, every site in the reservoir has pressure and saturation values for both the fractures and the matrix. In a dual-porosity model, flow occurs in fractures, and the rock matrix provides storage facility. Interconnected fractures facilitate the flow pathway to injection and production wells, which are presumed inside the fracture domain. In the dual-porosity model, the matrix and fracture domains are interconnected via an exchange term that associates each fracture cell with its matching matrix cell inside a grid block. Kazemi et al.^23^ expanded the Warren and Root methodology to multiphase flow and developed dual-porosity models for modelling fluid dynamics in naturally fractured reservoirs. Since then, the dual-porosity methodology has been employed in numerous studies for field-scale non-fossil resource predictions. The dual-porosity model is an essential depiction of a geologically intricate reservoir under these assumptions. As a result, significant work has been expended to enhance the accuracy of the dual-porosity model. Significant advancements encompass the subdomain method^24^, the multiple interacting continua method^25^, and procedures for pseudo capillary pressure and relative permeability^26^. Furthermore, dual-permeability models have been formulated that employ the same methodology as dual-porosity models but are enhanced by matrix-to-matrix flow^26^.

Outcrop characterization studies indicate that natural fractures exhibit significant variability in height, length, aperture, spacing, and network connectivity^27,28^, thereby underscoring a considerable divergence between actual conditions and the uniformity assumed in dual-porosity models. Consequently, DFMs have been developed to minimize the non-physical abstractions in dual-continuum models. Most DFMs utilise unstructured grids to depict a fracture network directly. In comparison to dual-porosity models, DFMs have multiple advantages. They can replicate actual fracture system geometry; thus, they explicitly consider the impact of individual fractures on fluid flow. Grid-defined fracture geometry limitations do not excessively limit them, so the fracture model is readily adaptable and updatable. The specification of the interaction between matrix and fracture is more direct, as it is contingent upon fracture geometry. One major problem with DFMs is that they often lead to systems of equations that are not continuous and have a complicated structure, which makes them hard to solve numerically. Baca et al.^29^ employed a Finite Element Method (FEM) to model the flow in fractured reservoirs with only one phase. Later, Kim and Deo^30^ improved the finite element approach for two-phase flow in fractured reservoirs. Current finite element based approaches work well for single-phase flow but don’t guarantee local mass conservation for multiphase flow in highly heterogeneous reservoirs. Hoteit and Firoozabadi^31^ employed a combination of finite element and discontinuous Galerkin methods to tackle this problem. Matthai et al.^32^ also used FEM for multiphase and incompressible flow in a porous medium with discrete fractures. Karimi-Fard et al.^33^ developed a DFM that works well for multiphase flow reservoirs. They did this by using an unstructured control volume finite difference formulation. An unstructured discretization technique is frequently needed to portray a fractured porous medium’s complexity accurately. All the DFMs listed above use unstructured gridding to appropriately show the shape of fracture networks.

While using CFD for CO_2_-EOR can provide us with useful information on the complex multiphase flow physics of CO_2_ injection in fractured reservoirs, the costs associated with numerical computations is exorbitant, which at field-scale becomes prohibitive^34^. For analysing the effects of various operational parameters of CO_2_-EOR, a significant amount of computational time is required, which limits the use of CFD for practical purposes where real-time data analysis is needed. Machine Learning (ML) based surrogate models offer a viable solution to this problem. Trained on CFD simulations outputs, ML models provide real-time predictions across a wide range of parameters while capturing key flow physics through the trained dataset^35,36^. In the context of CO_2_-EOR, recent studies show that ML based models can efficiently estimate oil recovery and sequestration performance using geological and operational data^37^. Operator-learning frameworks significantly reduce computational costs in reservoir-scale multiphase flow simulations. By integrating key flow physics via the training dataset, surrogate modelling allows rapid analysis, enabling closed-loop optimisation of CO_2_-EOR injection strategies^38^.

While most of the published studies on CO_2_-EOR focus on CO_2_ saturation, occurrence and impacts of breakthrough, and the evolution of pressure, only a few studies provide insights into CO_2_ front propagation and its injection rate to estimate storage capacity of fractured reservoirs. In this work, we present for the first time, a detailed spatio-temporal analysis of CO_2_ front propagation in a naturally fractured reservoir. To the authors’ best knowledge, Pore Volume (PV) resolved saturation evolution for CO_2_ front propagation analysis is not reported in published literature. This novel information not only highlights the challenges associated with CO_2_ injection in tight reservoirs, it also enables to identify the optimal and most practical CO_2_ injection strategy associated with CO_2_-EOR. Using this data, we develop a novel CFD-ML framework where CFD generated dataset is used to train a surrogate model for fast and accurate prediction of CO_2_ saturation within the reservoir. The combination of CFD’s accuracy in predicting complex multiphase flow physics and the speed of ML surrogate models, this study provides a unique demonstration of an efficient and practical methodology for real-time optimisation and decision making in CO_2_-EOR projects.

## Numerical modelling of CO_2_-EOR in naturally fractured reservoirs

This study employs Discrete Fracture Modelling (DFM) to investigate how CO_2_ is being transported and stored in naturally fractured rocks containing oil. The model accurately resolves complex multiphase flow behaviour between CO_2_ and oil and captures the critical interactions between fracture and the surrounding rock matrix. By including fracture-matrix dynamics, the method improves our understanding of flow distribution, CO_2_ migration pathways and overall reservoir performance. This information helps choose the best injection strategies, store CO_2_ and extract oil from naturally fractured reservoirs. The study extends the DFM modelling by integrating a Porous Discontinuity Model (PDM) similar to that proposed by Hawez et al.^39^, who implemented it in case of two-phase flow inside carbonate reservoirs. This method helps accurately model fluid flow at the fracture-matrix interface. This is where capillary pressure (P_c_), relative permeability (k_r_) and saturation (S) gradients vary significantly. In contrast to conventional models that depend on empirical or simplified transfer functions, PDM explicitly assesses P_c_ at the interface through a piecewise-defined function. This function is beneficial for cases where capillary forces prevail in the flow regime, particularly in low-permeability areas. This boundary condition of P_c_ is mathematically expressed as:

1 $$\:{{\mathrm{P}}^{\mathrm{l}}}_{\mathrm{c}{\upbeta\:}}\left({\mathrm{S}}_{{\upbeta\:}}^{\mathrm{l}}\right)=\left\{\begin{array}{c}{{\mathrm{P}}^{\mathrm{l}}}_{\mathrm{e}\mathrm{c},{\upbeta\:}}\:\:\:\:\:\:\:\:\:\:\:\:\:\:\:\:\:\:\:\:if\:\:\:\:\:\:\:\:\:\:\:\:\:{{\mathrm{P}}^{\mathrm{h}}}_{\mathrm{c}{\upbeta\:}}\left({\mathrm{S}}_{{\upbeta\:}}^{\mathrm{h}}\right)\le\:{{\mathrm{P}}^{\mathrm{l}}}_{\mathrm{e}\mathrm{c},{\upbeta\:}}\:\\\:{{\mathrm{P}}^{\mathrm{h}}}_{\mathrm{c}{\upbeta\:}}\left({\mathrm{S}}_{{\upbeta\:}}^{\mathrm{h}}\right)\:\:\:\:\:\:\:\:\:\:\:if\:\:\:\:\:\:\:\:\:\:\:{{\mathrm{P}}^{\mathrm{h}}}_{\mathrm{c}{\upbeta\:}}\left({\mathrm{S}}_{{\upbeta\:}}^{\mathrm{h}}\right)>{{\mathrm{P}}^{\mathrm{l}}}_{\mathrm{e}\mathrm{c},{\upbeta\:}}\end{array}\right.$$

where $\:{{\mathrm{P}}^{\mathrm{l}}}_{\mathrm{c}{\upbeta\:}}$ denotes the capillary pressure on the low-permeability side (i.e. the matrix), and $\:{{\mathrm{S}}^{\mathrm{l}}}_{\mathrm{c}{\upbeta\:}}$ is the corresponding phase saturation. Similarly, $\:{{\mathrm{P}}^{\mathrm{h}}}_{\mathrm{c}{\upbeta\:}}$ and $\:{\mathrm{S}}_{{\upbeta\:}}^{\mathrm{h}}$ represent the capillary pressure and saturation on the high-permeability side (i.e. the fracture). The parameter $\:{{\mathrm{P}}^{\mathrm{l}}}_{\mathrm{e}\mathrm{c},{\upbeta\:}}$ refers to the entry capillary pressure required to initiate CO_2_ propagation from the fracture into the matrix. The piecewise condition of Eq. (1) ensures that fluid cannot enter the matrix from the fracture unless P_c_ > $\:{{\mathrm{P}}^{\mathrm{l}}}_{\mathrm{e}\mathrm{c},{\upbeta\:}}$. As a result, the model reflects the physical reality of capillary barriers that prevent or delay fluid transfer between regions of different permeabilities.

Assuming the fracture initial capillary pressure is less than the rock matrix, the numerical model’s initial condition indicates an immobile fluid phase within the matrix. An inadequate driving force constrains this phase to surpass the entry pressure. Therefore, the phase saturation in the matrix starts at its residual value, suggesting that no sufficient flow occurs until the capillary pressure threshold is met. Particularly when modelling the earliest phases of injection or production, this precise representation of residual saturation and delayed imbibition (or drainage) provides a significant advantage over traditional methods. Using this enhanced approach, this study accurately handles complicated multiphase flow patterns in fractured reservoirs. Explicitly considering fracture geometry and capillary threshold effects helps better forecast fluid migration, pressure distribution and oil recovery efficiency under varying operating conditions. This is especially relevant in modern reservoir engineering applications like EOR and CO_2_ geosequestration where knowledge of the precise interaction between fluids and the rock matrix is essential for performance optimisation and long-term storage security. Including PDM in the DFM framework marks a significant advancement in the numerical modelling approach for fractured porous systems.

Advanced Computational Fluid Dynamics (CFD) based solvers have been employed to solve the numerical model. For this purpose, a commercial CFD package COMSOL Multiphysics has been chosen for its supervisor performance in predicting CO_2_ geosequestration and Enhanced Oil Recovery (EOR) in reservoirs over a fairly long time period; more than 12 h in the present study. Three models available in COMSOL Multiphysics have been employed in the present study. These are the Transport Model solving for mass and momentum conservation, the Multiphase Model in which Volume of Fluid (VOF) approach has been used, and the Porous Media Model solving for the Darcy equation.

### Geometric model and physical properties of the reservoir

**Fig. 1:**
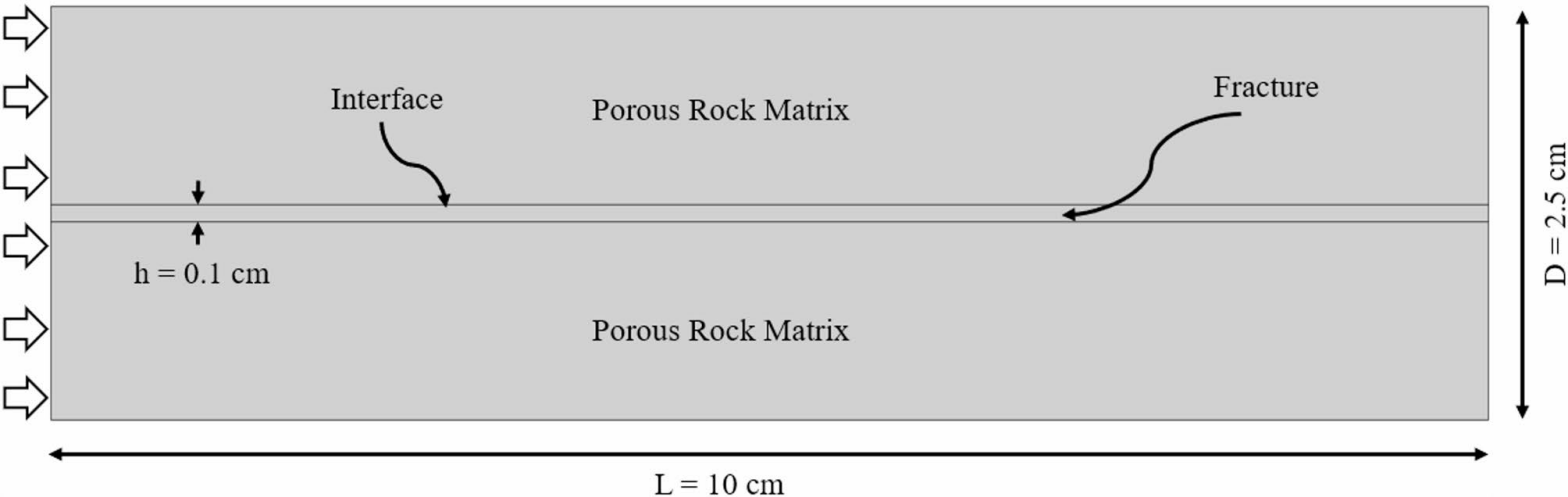
Geometric model of the fractured rock sample.

In order to ensure the validity of the results presented later in this study, the geometric model of fractured rock sample considered in the present study is the same as experimentally investigated by Zhang et al.^10^. Figure 1 depicts the two-dimensional geometric model of the DFM model of the rock sample, which is derived from low-permeability sandstone cores from a specific block in Changqing Oilfield. The core diameter (D) of the sample is 2.5 cm and its length (L) is 10 cm. The fracture region has been modelled as a rectangular channel with an aperture size of (h) of 0.1 cm, in-line with the experimental investigations of Zhang et al.^10^. A single planar fracture embedded in the porous media approach has been adopted to focus on the mechanistic phenomena at the fracture matrix interface and capillary entry control. This approach is widely used in both experimental and numerical studies^40–44^ as it offers conceptual clarity and computational traceability. CO_2_ injection into the sample takes place at the left-hand side boundary of the sample, while the exit/outlet is at the right-hand side boundary. In-line with the investigations carried out in Zhang et al.^10^, three different CO_2_ injection rates have been considered, as summarised in Table 1. These injection rates correspond to low (0.1 mL/min), intermediate (1 mL/min) and high (4 mL/min). CO_2_ is considered to be injected under Minimum Miscibility Pressure (MMP) and thus the CO_2_ displacement is immiscible, isolating the hydrodynamic effects independent of compositional mixing. For further accuracy, the porosity, permeability and initial oil saturation in the sample has been kept the same as experimentally measured in Zhang et al.^10^. The fracture has a permeability of 3.454 × 10^5^ mD. The oil is a blend of degassed crude oil and aviation kerosene in a 1:6 ratio, resulting in a light oil with a density of 857 kg/m³ and a viscosity of 13.29 mPa·s at 50 °C.

**Table 1:** Physical properties of the rock sample for different CO_2_ injection rates.

Condition	Porosity (%)	Permeability (mD)	Oil Saturation (mL)	CO_2_ Injection Rate (mL/min)
1	18.52	5.02	6.58	0.1 (Low)
2	17.98	4.86	6.91	1.0 (Intermediate)
3	18.21	5.42	6.59	4.0 (High)

### Mesh independence testing

A structured quadrilateral mesh has been generated in the flow domain to minimize numerical errors and improve prediction accuracy^45^. In order to ascertain that the accuracy of the numerical predictions is independent of the mesh element sizing, five different mesh schemes have been generated in the flow domain. Element size in the fracture aperture is kept considerably smaller than in the rock matrix to ensure accurate characterization of complex flow behaviour at the fracture-matrix interface. Oil Recovery Factor (RF) is the chosen evaluation parameter for mesh independence testing (and for validation in the next section), as in Zhang et al.^10^. Similarly, CO_2_ is injected till 50% of injected Pore Volume (PV) to later compare the numerical results with the published experimental results. Here, the pore volume (PV) is defined as:

1 $$\:\mathrm{P}\mathrm{V}={\upphi\:}\:\times\:{\mathrm{V}}_{\mathrm{b}\mathrm{u}\mathrm{l}\mathrm{k}}$$

where φ is the porosity and V_bulk_ is total volume of the core.

**Fig. 2:**
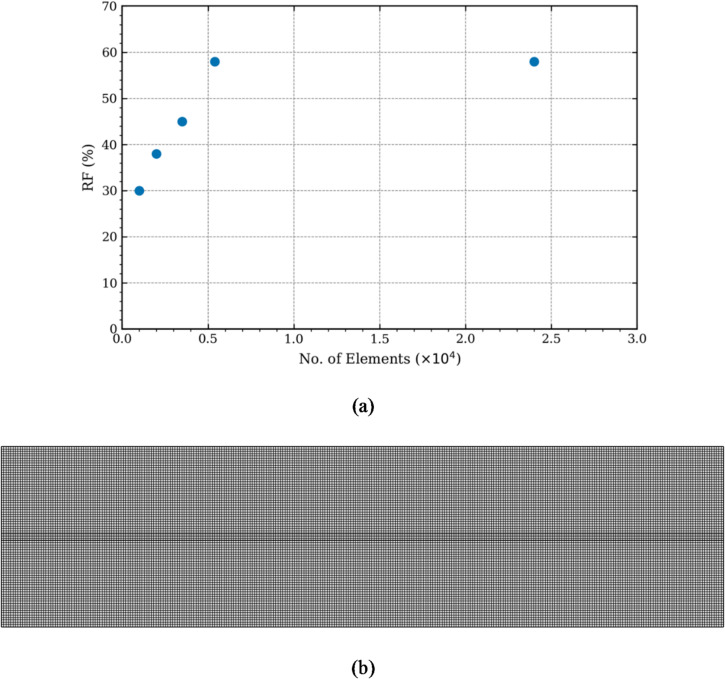
(**a**) Mesh independence test results and the (**b**) selected mesh.

The five different mesh schemes range from 0.1 × 10^4^ to 2.4 × 10^4^ total number of mesh elements. Figure 2(a) depicts the results of the mesh independence testing where it can be clearly seen that the cumulative oil RF increases (from 30% to 58%) as the total number of mesh elements increase (from 0.1 × 10^4^ to 0.54 × 10^4^). Further increasing the number of mesh elements to 2.4 × 10^4^ does not change oil RF suggesting that both the meshes with 0.54 × 10^4^ and 2.4 × 10^4^ are capable of accurately characterizing CO_2_-EOR in fractured reservoirs. To be on the safe side, the mesh with 2.4 × 10^4^ has been chosen for further analysis. This mesh consists of 78 elements specified in the radial direction (y-direction), and 300 elements in the axial direction (x-direction), as shown in Fig. 2(b).

## Characterisation of CO_2_-EOR in naturally fractured reservoirs

As CO_2_-EOR is a simultaneous dual process where CO_2_ replaces oil in fractured reservoirs, its characterisation is also carried out in a dual manner. In the following sections, after validating the numerical results against the experimental data of Zhang et al.^10^, injection and geosequestration of CO_2_ is discussed in detail, followed by the analysis of reservoir’s constitutive relationships i.e. capillary pressure and relative permeability.

### Numerical model validation

**Fig. 3:**
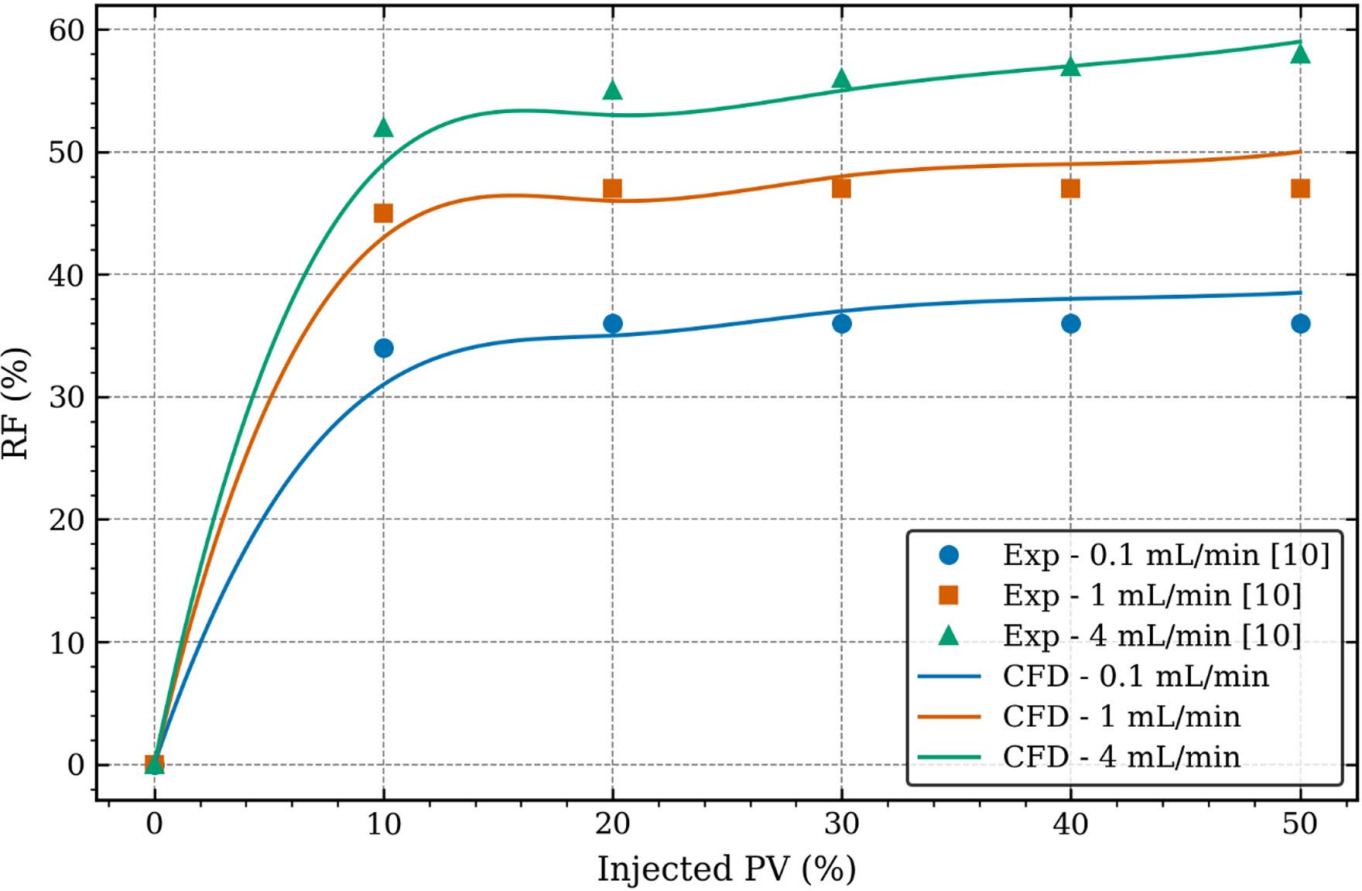
Numerical model validation against the experimental data.

Figure 3 depicts the variations in the oil Recovery Factor (RF) against the injected Pore Volume (PV) for three different CO_2_ injection rates considered in Zhang et al.^10^ and summarised in Table 1. Here the oil RF refers to how much oil has been extracted from the reservoir by injecting CO_2_, while injected PV refers to how much CO_2_ is injected as a percentage of pore (void) volume available in the reservoir, which is dictated by the reservoir porosity as in Table 1. The markers represent the experimental data obtained from Zhang et al.^10^ while the solid curves represent the CFD data from the present study. It can be seen that the general trend in the experimental data is that as injected PV increases from 0% to 10%, oil RF increases substantially, while further increase in injected PV shows very small change in oil RF especially at low injection rates. As CO_2_ injection rate increases, oil RF also increases, almost proportionally, and at higher injection rates, gradual increase in RF is observed till injected PV of 50%.

The numerically predicted results show the same trend for all different CO_2_ injection rates. At injected PV of 10%, CFD underpredicts oil RF at all injection rates. The average difference between experimental data and CFD results considering all injection rates at injected PV of 10% is 7%. At injected PV of 50%, CFD overpredicts oil RF at all injection rates by 4.3% on average considering all injection rates. It is also noteworthy that CFD predicted oil RF matches more closely with experimental data at higher injection rates compared to lower injection rates. As the difference in the final oil RF (at injected PV of 50%) is less than 5%, the CFD results of the present study are considered accurate here and thus suitable for further investigations and analyses.

### CO_2_ geosequestration

**Fig. 4:**
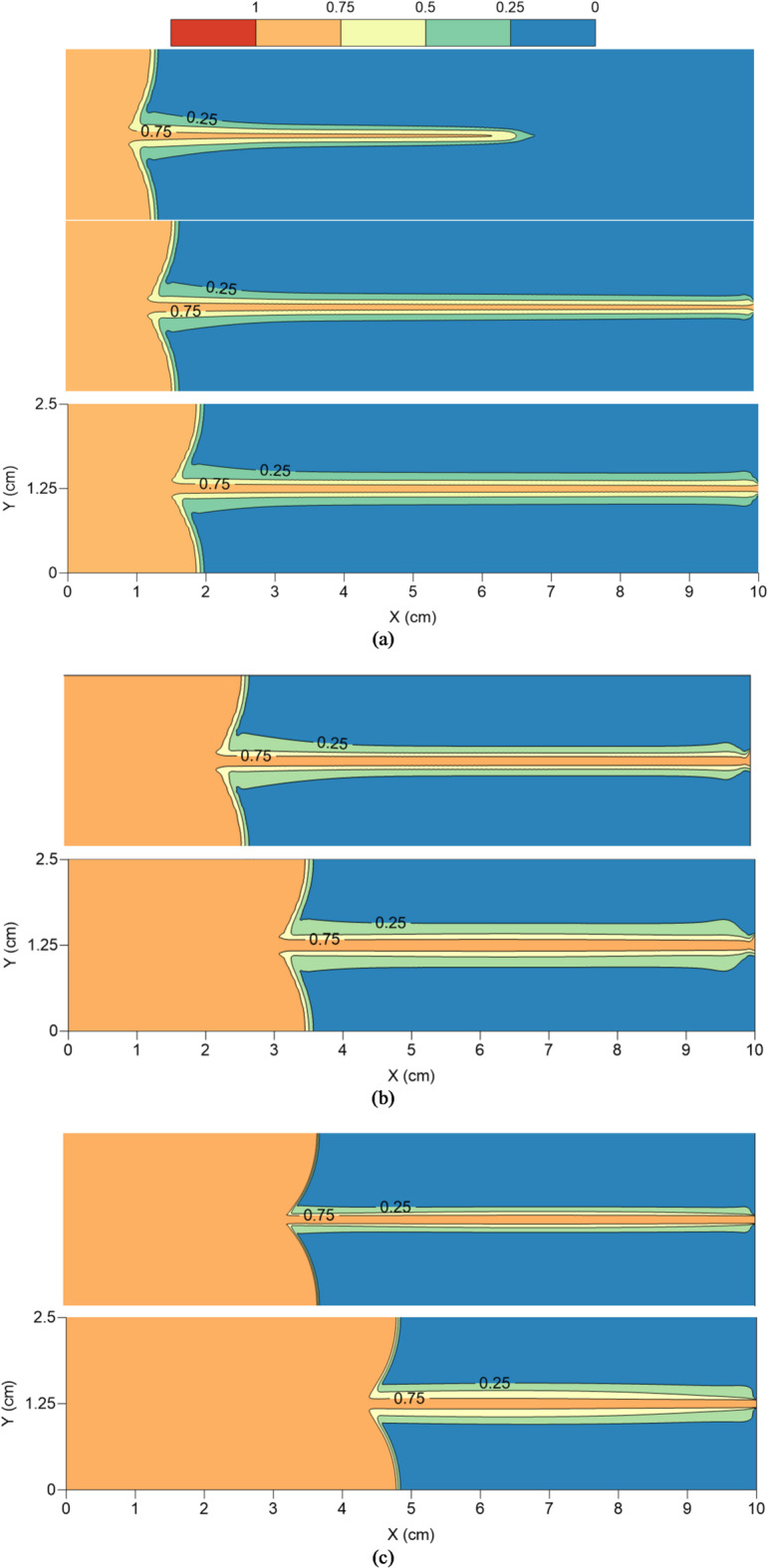
Variations in the saturation of CO_2_ in the fractured rock sample for different injection rates and at different injected PV values. (**a**) CO2 saturation at 10%, 30% and 50% injected PV for 0.1 mL/min injection., (**b**) CO2 saturation at 10% and 50% injected PV for 1 mL/min injection., (**c**) CO2 saturation at 10% and 50% injected PV for 4 mL/min injection.

Figure 4 depicts the variations in CO_2_ saturation (S_CO2_) within the fractured rock sample for different injection rates considered in the present study. Figure 4(a) corresponds to injection rate of 0.1 mL/min (low), Fig. 4(b) to 1 mL/min (intermediate) and Fig. 4(c) to 4 mL/min (high). Each figure further depicts S_CO2_ at different injected PV values, thus demonstrating how CO_2_ propagates into the fractured rock. In case of low injection rate, S_CO2_ variations are shown at injected PV values of 10%, 30% and 50%, while for intermediate and high injection rates, CO_2_ saturation is shown at 10% and 50% injection rates only.

Figure 4(a) depicts that during the initial phase of CO_2_ injection (injected PV = 10%), CO_2_ propagates much further along the fractured zone compared to the rock matrix, as expected due to lower permeability of the matrix. However, the breakthrough hasn’t occurred yet and thus, there is negligible preferential propagation of CO_2_ from the fracture into the matrix. As the injected PV increases to 30%, breakthrough can be seen to occur while the CO_2_ front in the matrix region has also advanced. Advancing in time as the injected PV increases to 50%, only slight advancement in CO_2_ front is observed in the matrix, which is consistent with the results presented in Fig. 3. Thus, at low injection rates, capillary forces dominate viscous forces in the flow domain due to higher capillary entry pressure, leading to capillary fingering and reduced sweep efficiency, a trend also observed by Dullien^46^. With increase in the injection rate, breakthrough is observed sooner, at injected PV of 10%, but as the injection rates are higher, CO_2_ front in the matrix has also advanced proportionally. At intermediate injection rate in Fig. 4(b), a balance between the capillary and viscous forces is evident through the occurrence of preferential flow from the fracture into the matrix, resulting in higher CO_2_ saturation in the reservoir, and thus higher oil RF (Fig. 3). This is in-line with the Buckley-Leverett theory^47^ which shows how fractional flow and saturation are affected by the capillary and viscous forces. At high injection rate of Fig. 4(c), viscous forces dominate, decreasing the impact of capillary trapping and demonstrating a piston-like displacement front, which is also reported by Krevor et al.^48^.

Low and high injection rates have their own advantages and disadvantages. High injection rates lead to higher S_CO2_ and oil RF but the breakthrough is premature leading to inefficient long-term CO_2_ trapping^49^. At low injection rates, capillary trapping is elevated for long-term CO_2_ storage however, the injection process becomes less practical for industrial-scale deployment. An intermediate injection rate might be the most sensible option which can be further optimised to balance capillary and viscous forces to maximise both CO_2_ geosequestration and EOR^50,51^.

**Fig. 5:**
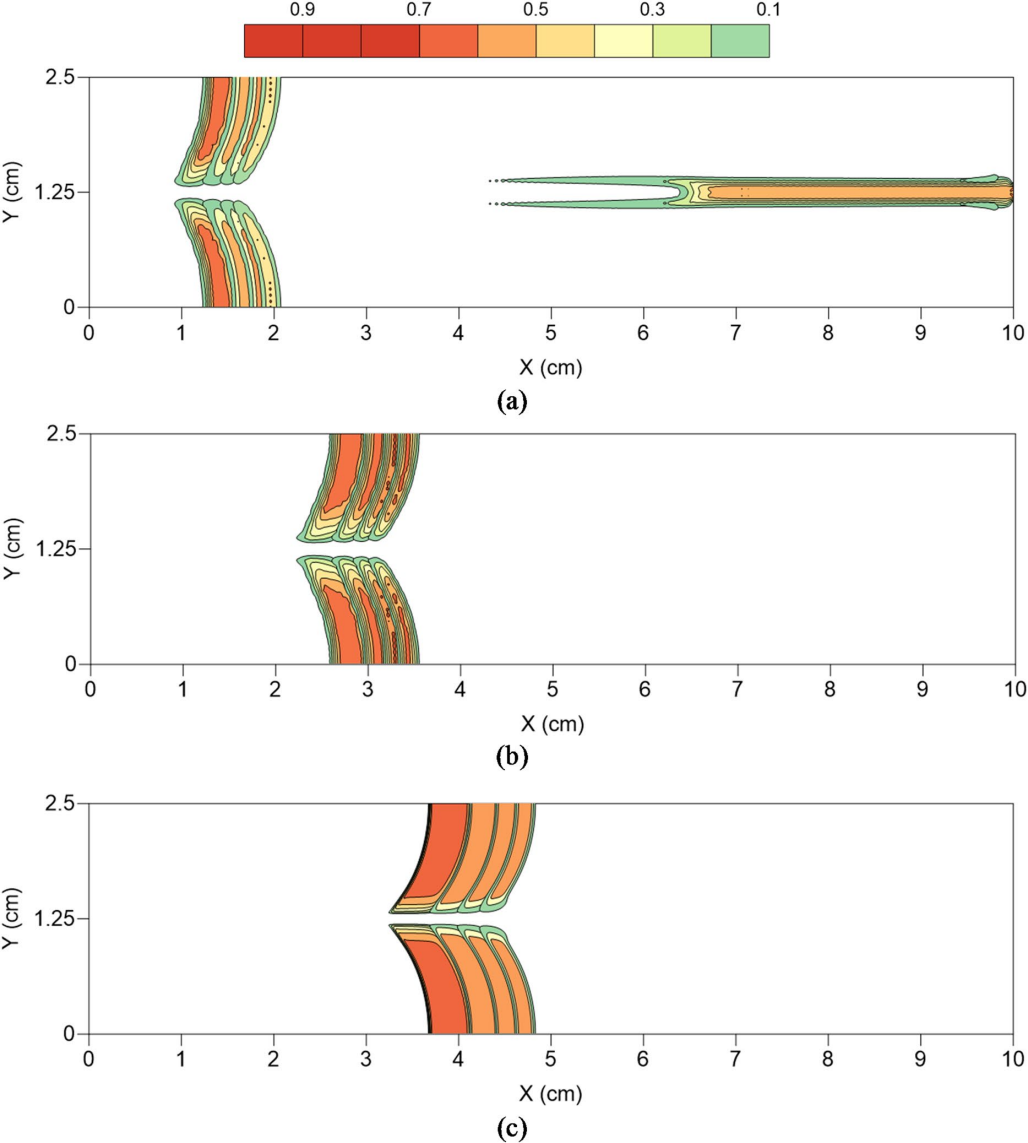
Difference in CO_2_ saturation between consecutive injected PV values for different injection rates. (**a**) Low injection, (**b**) Intermediate injection, (**c**) High injection.

CO_2_ geosequestration, as opposed to EOR, is a relatively new technology that normally lacks detailed understanding of CO_2_ dynamics when it comes to the propagation of CO_2_ in the reservoir. In the present study, a novel attempt has been made to explicitly investigate, analyse and characterise CO_2_ propagation in naturally fractured reservoirs. Figure 5 depicts the difference in S_CO2_ variations as injected PV increases, for the different injection rates considered. Here the difference in S_CO2_ variations refers to $\:{{\mathrm{S}}_{\mathrm{C}\mathrm{O}2}}_{(\mathrm{n}+1)}-{{\mathrm{S}}_{\mathrm{C}\mathrm{O}2}}_{\left(\mathrm{n}\right)}$. Thus, Fig. 5(a) shows 4 layers of CO_2_ front advancing where layer 1 (left most), which also includes the variations seen in the fracture region from X = 4.2 cm till the outlet boundary, refers to S_CO2_ at injected PV of 20% minus S_CO2_ at injected PV of 10%. Similarly, layer 2 (right of layer 1), is for injected PV of 30% minus injected PV of 20%. Layer 4 (right most) is for injected PV of 50% minus injected PV of 40%. Hence, layers 1 to 4 depict how CO_2_ front is advancing in the reservoir at low injection rate. Figures 5(b) and 5(c) show the same but for intermediate and high injection rates respectively. Figure 5 shows three important features. Firstly, breakthrough at low injection rates in slower than at intermediate and higher injection rates, which is evident from the contours present in the fracture zone of Fig. 5(a). Secondly, higher injection leads to further displacement of CO_2_ front and hence, higher CO_2_ geosequestration and oil RF. Both these features are, to some extent, also visibly in Fig. 4. The third and the most important feature observed in Fig. 5 is the thickness of individual layers and how it changes as injection rate increases. Closely monitoring Fig. 5(a) reveals that layer 1 (left most) is wider (in X direction) compared to layer 2. Similarly, layer 2 is wider than layer 3 and layer 3 is wider than layer 4, which is the narrowest of all four layers. This clearly indicates slowing down of CO_2_ front as it occupies more volume in the rock, and thus also explains why the oil RF in Fig. 3, after rapid initial increase, slows down as injected PV increases. It is also noteworthy here that not only does the layers become thinner as injected PV increases, change in S_CO2_ also decreases (higher contour values in the central zone and more gradients in layer 1 compared to layer 4). It can be seen in Fig. 5(b) for intermediate injection rate that these layers are wider than in Fig. 5(a), and layer 4 has higher S_CO2_ values than low injection rate layer 4, while the rate of change of S_CO2_ is seen to reduce as injected PV increases. In case of high injection rate in Fig. 5(c), the layers become even wider and are mostly dominated by the central high S_CO2_ value regions. Interestingly, although layers become thinner as injected PV increases, the rate of change of S_CO2_ remains almost constant, unlike in case of low injection rate, and also in intermediate injection rate to some extent. This is the main reason behind increase in oil RF in Fig. 3 for intermediate and high injection rates from injected PV 20% onwards.

**Fig. 6:**
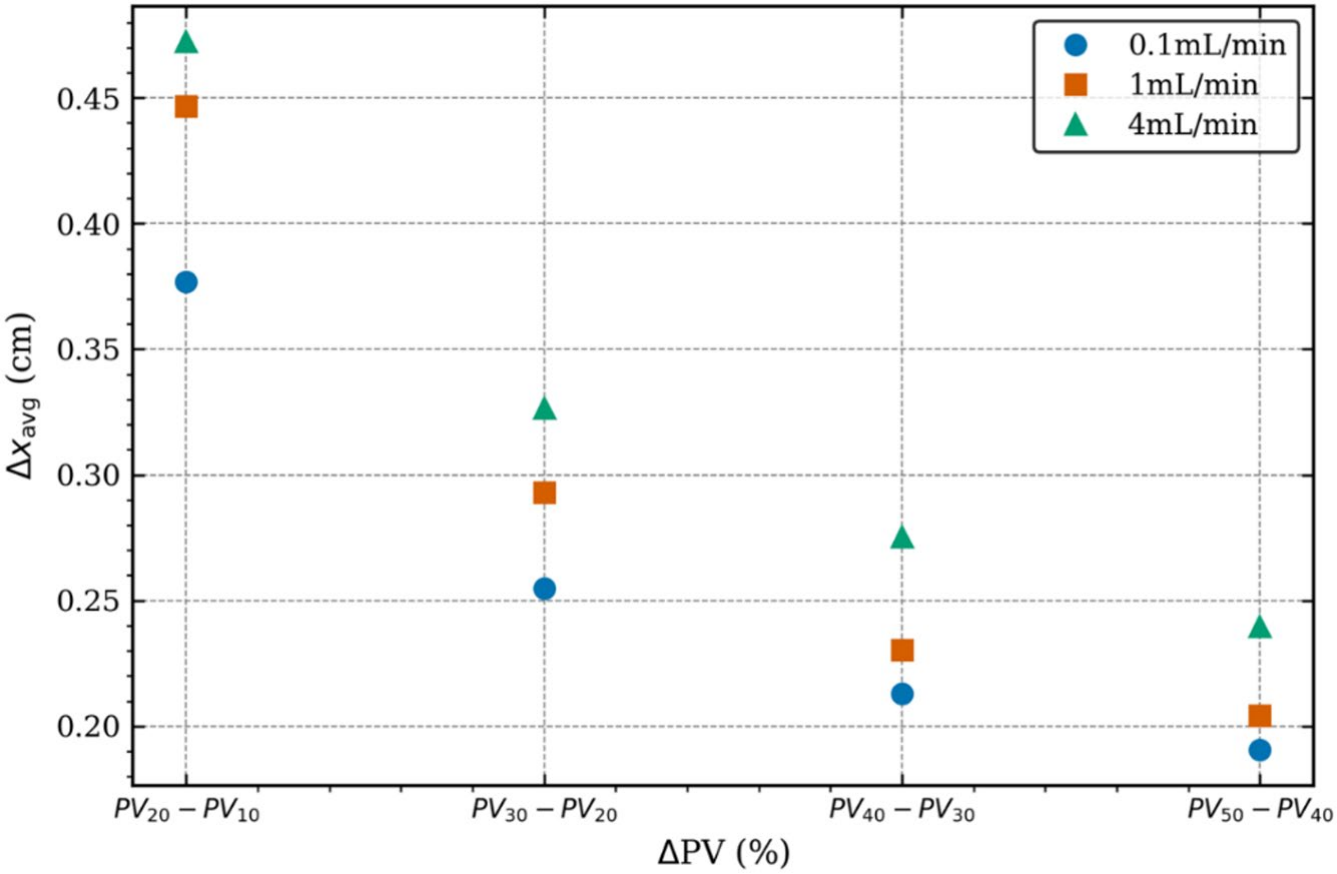
Axial propagation of CO_2_ front in the fractured rock sample at different injection rates.

Quantifying the results of Figs. 5 and 6 shows how CO_2_ front advances axially as it is being injected into the rock sample at different injection rates. Y-axis shows how much CO_2_ front moves forward on average considering the radial asymmetry in the layers, while X-axis shows the difference in injected PV values, same as the different layers in Fig. 5. Decrease in the rate of CO_2_ front advancement is obvious for all injection rates. At low injected PV values (10%), intermediate injection rate is closer to high injection rate, while at higher injected PV values (50%), it is closer to the low injection rate. Thus, for intermediate injection rate, CO_2_ saturation in the rock sample increases sharply initially, enabling faster CO_2_ geosequestration. At higher injected PV, although the propagation of CO_2_ front has slowed down, for actual saturation values, there is a need to draw the curves showing the variations in S_CO2_.

**Fig. 7:**
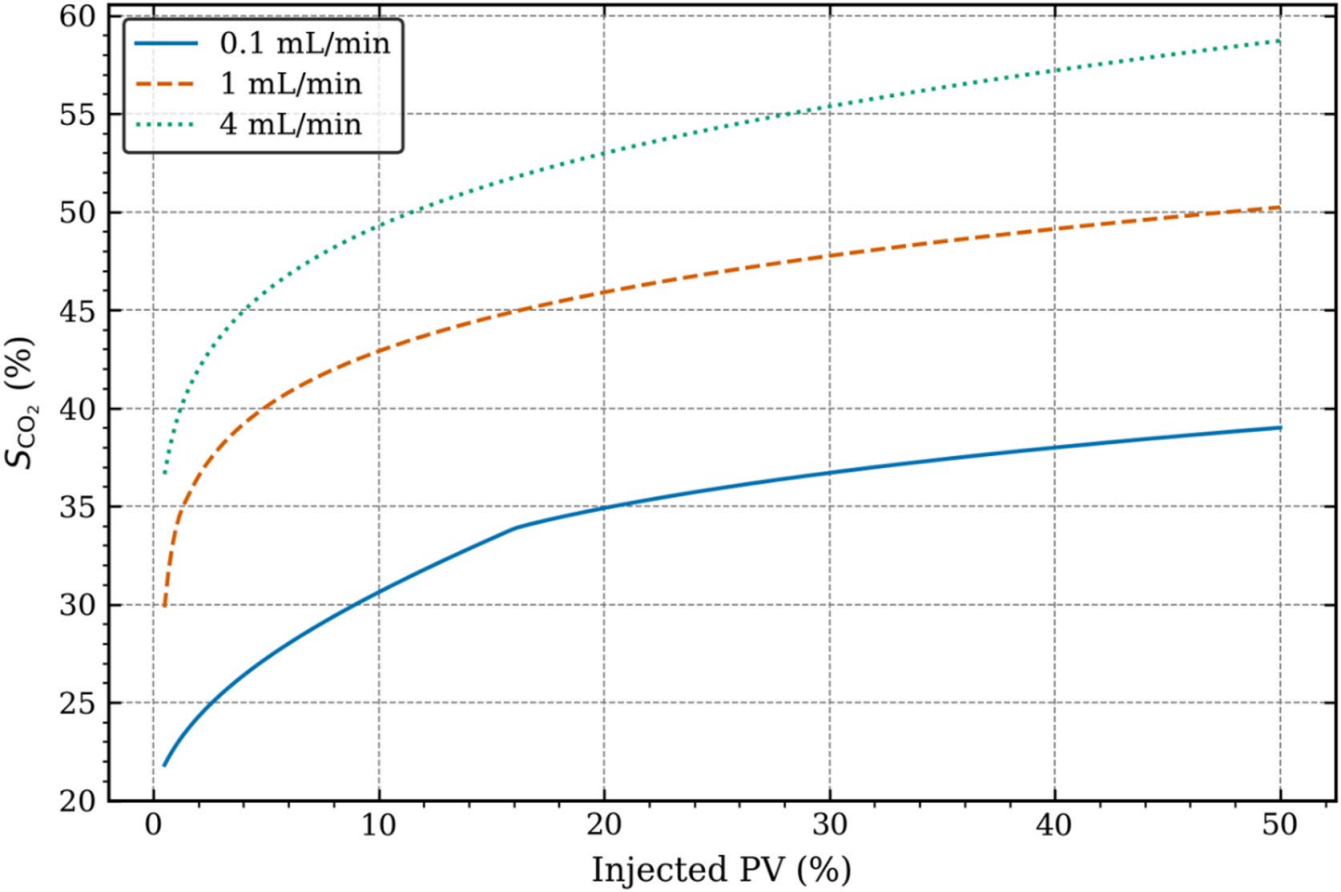
CO_2_ saturation variations against injected PV at different injection rates.

Figure 7 depicts the variations in CO_2_ saturation (S_CO2_) in the fractured rock sample as injected PV increases, for the different injection rates considered. The initial sharp increase in S_CO2_ is evident from all three curves, which is in-line with higher Δx_avg_ values in Fig. 6 and oil RF in Fig. 3. Analysing the intermediate injection curve, it can be seen that the actual saturation doesn’t drop such that it comes closer to low injection rate curve, rather we see a steady increase in S_CO2_ at higher injected PV values. The question arises then how come CO_2_ front propagation has slowed down. In order to answer this question, we refer back to Fig. 4 and the discussion regarding the Buckley-Leverett theory^47^ i.e. preferential flow from the fracture zone into the rock matrix is observed. Thus, although the rate of propagation of CO_2_ front has decreases (Fig. 6), CO_2_ geosequestration continues to increase (Fig. 7) as CO_2_ propagates radially across the fracture-matrix interface. This is also observed in case of high injection rate but the preferential flow is limited due to the injection pressure, pushing CO_2_ through the fracture zone due to strong viscous force. This theory is supported by Bennion et al.^52^, Akbarabadi and Piri^53^ and Chadwick et al.^54^, who have shown how high injection rate reduces residual trapping^16^, low injection rate leads to greater capillary heterogeneity^55^, while optimal injection balances plume distribution and trapping efficiency^56^, which in the current study matches with the intermediate injection rate.

### Capillary pressure and relative permeability

**Fig. 8:**
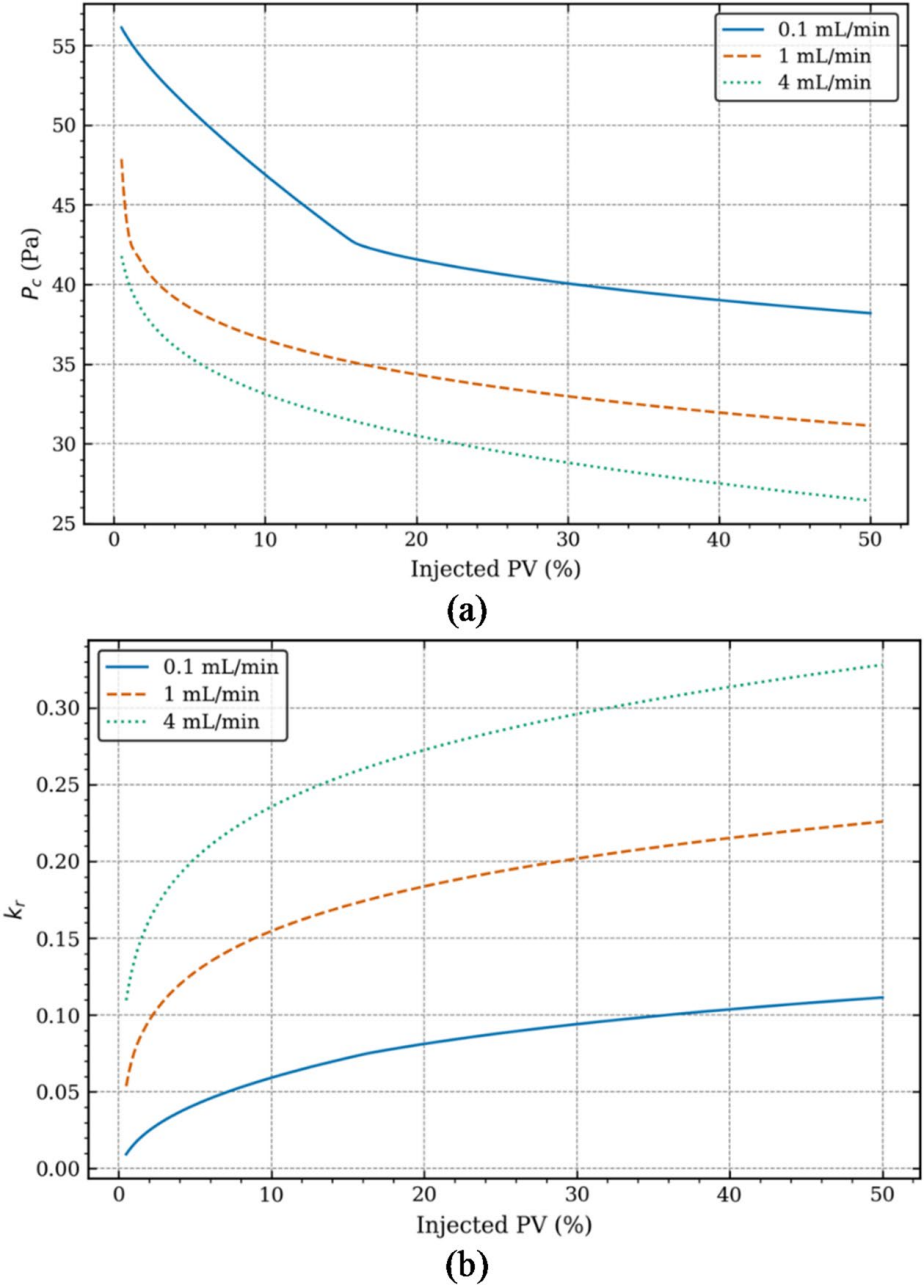
Variations in (**a**) capillary pressure and (**b**) relative permeability with injected PV at different injection rates.

As mentioned in Section “Characterisation of CO_2_-EOR in naturally fractured reservoirs”, in order to complete the discussion on EOR, we also analyse the effect of CO_2_ injection rates on the Capillary Pressure (P_c_) and Relative Permeability (k_r_). Figure 8 shows how P_c_ and k_r_ varies with respect to injected PV at different injection rates. Starting with discussions over P_c_ in Fig. 8(a), it can be seen that P_c_ initially drops sharply, followed by a more gradual decrease. In case of low injection rate, the initial drop is from 56 Pa to 42 Pa at 20% PV, indicating good sweep efficiency, though higher values of P_c_ clearly indicate dominance of capillary forces, which leads to slower but more stable CO_2_ geosequestration, while CO_2_ propagation primarily occurs in the fracture zone. High injection rate starts with much lower P_c_ of 42 Pa which drops to 31 Pa at 20% PV, indicating fast CO_2_ storage but at the expense of poor sweep efficiency due to early breakthrough. Thus, the potential of viscous fingering is very high which negatively impacts EOR. In case of intermediate injection, the initial drop in P_c_ from 48 Pa at 0% PV to 34 Pa at 20% PV indicates good sweep efficiency and balance between low and high injection rates. Early CO_2_ propagation and storage is noticed along with preferential flow across the fracture-matrix interface. In terms of k_r_, as the injection rate and injected PV increases, k_r_ increases. Lower k_r_ for low injection rate indicates slower displacement of oil from the matrix as CO_2_ mostly stays in larger pores. For intermediate injection, capillary forces are reduced enough to allow CO_2_ to flow more easily through the matrix, enabling CO_2_ geosequestration. Higher injection rate curve with higher k_r_ values indicate that CO_2_ is being forced to flow through the fracture, raising CO_2_ saturation initially, but at the expense of poor sweep efficiency due to early breakthrough and thus, lower EOR.

## Development of machine learning models for CO_2_ geosequestration

High fidelity CFD modelling approach above helps understand the complex flow behaviour and storage of CO_2_ in naturally fractured rocks, however this modelling approach is computationally very expensive. For fast, yet accurate, prediction of CO_2_ storage in geological formations, two Machine Learning (ML) models have been developed and trained on the data obtained from CFD. Predictive capabilities of these ML models are then compared against the training data for validation purposes. Characterisation and comparison of these models is carried out to select the best ML model for characterising CO_2_ geosequestration. The following sections provide details of the ML models developed.

### Data processing

**Fig. 9:**
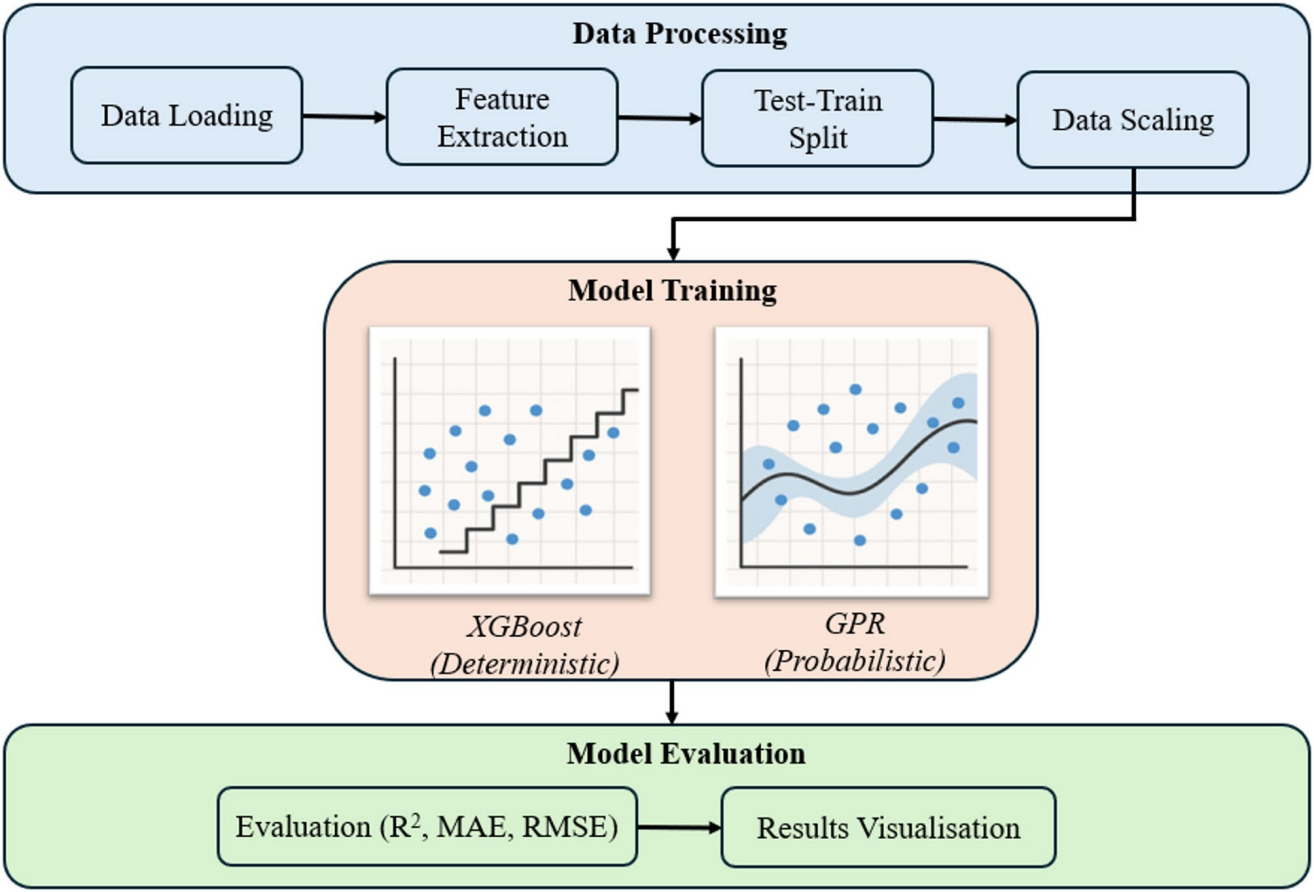
Machine learning model development and evaluation workflow.

It is important to establish the workflow adopted in this study for ML models development and evaluation. Figure 9 depicts the ML workflow where it can be seen that the application of ML is carried out in three stages i.e. data processing, model training, and model evaluation^57^. This section provides details of data processing stage. The data processing stage further comprises of, in this case, four steps, i.e.:

1. Data loading: CFD data from Fig. 7 is saved in numerical form and imported into Python environment, which is commonly used for developing surrogate models^58^.
2. Feature extraction: The primary input feature is the injected Pore Volume (PV) while the output feature (target) is the saturation of CO_2_ in the rock sample (S_CO2_). The injection rate of CO_2_ is the secondary input feature. Using numerical simulation data as features for supervised learning tasks is well established in flow through porous media studies^59^.
3. Test-train split: For each injection rate, the related dataset (input and target) is split into training and testing sets in 80:20 ratio. Model training was performed exclusively on the training data, with the unseen testing data reserved for evaluating model generalization. For the splitting process, random_state = 42 is used in order ensure that the results obtained are reproducible^60^.
4. Data scaling: Standardization of the target variable (S_CO2_)​​​​ is performed through the StandardScaler class in the scikit-learn library in Python. This transformation scales the data from 0 to 1 i.e. having zero mean and unit variance. The scaler was first applied only on the training segment of the data and then applied on both the training and testing sets to avoid information leakage, a well-established practice in ML^61^.

### Gaussian process regression

Gaussian Process Regression (GPR) is a non-parametric, Bayesian statistical approach applied for regression related problems^62^. Rather than parametric models that find a fit for a function with a fixed number of parameters, GPR creates a probability distribution over a space of functions^63^, thus it is a probabilistic model. A Gaussian Process (GP) is simply an infinite-dimensional generalization of a multivariate Gaussian distribution, completely characterized by a mean function m(x) and a covariance (or kernel) function k(x, x´)^64^. The kernel plays a critical role since it outlines the similarity between data points and records prior beliefs concerning the characteristics of the function like its smoothness or periodicity^63–65^. Given a training set $\:\mathrm{D}=\{\left({\mathrm{x}}_{\mathrm{i}},{\mathrm{y}}_{\mathrm{i}}\right){\}}_{\mathrm{i}=1}^{\mathrm{N}}$​​, with input vectors xi (injected PV) and scalar outputs yi​ (S_CO2_), GPR assumes that the observed outputs y are generated from a latent function f(x) drawn from a GP, typically with additive Gaussian noise $\:\mathrm{ϵ}\sim\:\mathcal{N}\left(0,{{\upsigma\:}}_{\mathrm{n}}^{2}\right)$, such that $\:\mathrm{y}=\mathrm{f}\left(\mathrm{x}\right)+\mathrm{ϵ}.$ The predictive distribution for a new input $\:{\mathrm{x}}_{\mathrm{*}}$ is also Gaussian, hence enabling both a mean prediction and a variance, which quantifies the uncertainty of the prediction.

For a new test input $\:{\mathrm{x}}_{\mathrm{*}}$​, the predictive mean $\:\stackrel{-}{{\mathrm{f}}_{\mathrm{*}}}$ and variance $\:\mathrm{v}\mathrm{a}\mathrm{r}\:\left({\mathrm{f}}_{\mathrm{*}}\right)$ are given by:

2 $$\:\stackrel{-}{{\mathrm{f}}_{\mathrm{*}}}=\mathrm{K}\left({\mathrm{x}}_{\mathrm{*}},\mathrm{X}\right){\left[\mathrm{K}\left(\mathrm{X},\mathrm{X}\right)+{{\upsigma\:}}_{\mathrm{n}}^{2}\mathrm{I}\right]}^{-1}\mathrm{y}$$

3 $$\:\mathrm{v}\mathrm{a}\mathrm{r}\left({\mathrm{f}}_{\mathrm{*}}\right)=\mathrm{K}\left({\mathrm{x}}_{\mathrm{*}},{\mathrm{x}}_{\mathrm{*}}\right)-\mathrm{K}\left({\mathrm{x}}_{\mathrm{*}},\mathrm{X}\right){\left[\mathrm{K}\left(\mathrm{X},\mathrm{X}\right)+{{\upsigma\:}}_{\mathrm{n}}^{2}\mathrm{I}\right]}^{-1}\mathrm{K}\left(\mathrm{X},{\mathrm{x}}_{\mathrm{*}}\right)$$

where X and y stand for the training inputs and outputs, respectively, ​K (X_A_, X_B_) refers to the matrix of kernel evaluations between all pairs of points from set X_A_​ and X_B_, and I is the identity matrix. Diagonal elements of $\:\mathrm{v}\mathrm{a}\mathrm{r}\:\left({\mathrm{f}}_{\mathrm{*}}\right)$ delivers the predictive variances for each test point, which are used in constructing confidence intervals^66,67^. The major strengths of GPR that relate to this study include its capability to estimate the uncertainty limits (confidence intervals) of its predictions as well as its robust performance, especially with smaller datasets, provided a proper kernel is chosen^66,68^. In the present study, a composite kernel comprising a ConstantKernel, a Radial Basis Function (RBF) kernel, and a WhiteKernel are chosen based on the expected behaviour of the target variable S_CO2_^69^. The RBF kernel defined as:

4 $$\:{\mathrm{k}}_{\mathrm{R}\mathrm{B}\mathrm{F}}\left(\mathrm{r};\mathrm{l},{{\upsigma\:}}^{2}\right)={{\upsigma\:}}^{2}\mathrm{exp}\left(-\frac{{\mathrm{r}}^{2}}{2{\mathrm{l}}^{2}}\right)$$

assumes the underlying function is infinitely smooth, which is quite a suitable assumption for CO_2_ saturation as a function of injection rate^69^. The defined kernel structure is:

ConstantKernel (1.0, constant_value_bounds = (1e-3, 1e3)) * RBF (length_scale = 1.0, length_scale_bounds = (1e-2, 1e3)) + WhiteKernel (noise_level = 0.01, noise_level_bounds = (1e-5, 1e1)).

Kernel hyperparameters (e.g., length scale l$\:,$ variance σ^2^, noise level σ^2^_n_​​) are optimized during the training process by maximizing log marginal likelihood. To avoid convergence to local optima, the optimiser (n_restarts_optimizer) is restarted 15 times, and the target variable is internally normalized by the GPR model^70^.

### Extreme gradient boosting

Extreme Gradient Boosting (XGBoost) is an advanced implementation of gradient boosting machines, which uses ensemble learning for building multiple decision trees one after another^71^. Every new tree is used to train the ensemble of the previously trained trees to correct its errors and thus, it is a probabilistic model^71^. XGBoost has an excellent predictive accuracy, computational efficiency, control model complexity and ways of avoiding overfitting^71,72^. The underlying concept of gradient boosting is related to additive building of the ensemble of weak learners (typically decision trees). For $\:{{\widehat{\mathrm{y}}}_{\mathrm{i}}}^{\left(\mathrm{t}-1\right)}$ being the prediction for the i^th^ sample after (t-1) iterations, a new tree ​​f_t_(xi) is added to minimise an objective function Obj^(t)^ that can be approximated through a second-order Taylor expansion of the loss function $\:\mathrm{L}\left({\mathrm{y}}_{\mathrm{i}},\widehat{{\mathrm{y}}_{\mathrm{i}}}\right)$:

5 $$\:\mathrm{O}\mathrm{b}{\mathrm{j}}^{\left(\mathrm{t}\right)}\approx\:{\sum\:}_{\mathrm{i}=1}^{\mathrm{N}}\left[\mathrm{L}\left({\mathrm{y}}_{\mathrm{i}},{{\widehat{y}}_{i}}^{\left(t-1\right)}\:\right)+{\mathrm{g}}_{\mathrm{i}}{\mathrm{f}}_{\mathrm{t}}\left({\mathrm{x}}_{\mathrm{i}}\right)+\frac{1}{2}{\mathrm{h}}_{\mathrm{i}}{\mathrm{f}}_{\mathrm{t}}^{2}\left({\mathrm{x}}_{\mathrm{i}}\right)\right]+{\Omega\:}\left({\mathrm{f}}_{\mathrm{t}}\right)$$

where $\:{\mathrm{g}}_{\mathrm{i}}={\partial\:}_{{{\widehat{\mathrm{y}}}_{\mathrm{i}}}^{\left(\mathrm{t}-1\right)}}\mathrm{L}\left({\mathrm{y}}_{\mathrm{i}},{{\widehat{\mathrm{y}}}_{\mathrm{i}}}^{\left(\mathrm{t}-1\right)}\right)$ and $\:{\mathrm{h}}_{\mathrm{i}}={{\partial\:}^{2}}_{{{\widehat{\mathrm{y}}}_{\mathrm{i}}}^{\left(\mathrm{t}-1\right)}}\mathrm{L}\left({\mathrm{y}}_{\mathrm{i}},{{\widehat{\mathrm{y}}}_{\mathrm{i}}}^{\left(\mathrm{t}-1\right)}\right)$ are the first and second order gradient statistics of the loss function. $\:{\Omega\:}\left({\mathrm{f}}_{\mathrm{t}}\right)$ is a regularisation term for the t^th^ tree that penalises its complexity, that is usually given by:

6 $$\:{\Omega\:}\left({\mathrm{f}}_{\mathrm{t}}\right)={\upgamma\:}{\mathrm{L}}_{\mathrm{T}}+\frac{1}{2}{\uplambda\:}{\sum\:}_{\mathrm{j}=1}^{{\mathrm{L}}_{\mathrm{T}}}{\mathrm{w}}_{\mathrm{j}}^{2}$$

where L_T_​ is the number of leaves in the tree f_t_, w_j_​ are the scores (weights) on each leaf, γ and λ are regularisation parameters which govern the penalty for tree complexity and leaf weight magnitude respectively. The objective function is minimised to get the structure of each tree ​f_t_(xi) as well as optimal leaf weights $\:{\mathrm{w}}_{\mathrm{j}}^{\mathrm{*}}$. Some promising benefits that make XGBoost a good choice for this study is its ability to capture complex non-linear relationships and the robustness to outliers, to a certain degree.

The XGBoost regressor was configured with the following hyperparameters:

- n_estimators = 300: The number of trees in the ensemble altogether.
- max_depth = 4: The maximum level of depth allowed for each individual tree to control the complexity of the tree.
- learning_rate (eta): Shrinkage parameter that shrinks the share of each tree to avoid overfitting. It is set to 0.1.
- subsample = 0.8: Fraction of training samples used to grow each tree.
- colsample_bytree = 0.8: Fraction of attributes used to grow each tree.
- early_stopping_rounds = 10: Training stops if performance does not improve on a validation set of 10 consecutive iterations.

### Model training and evaluation

Individual models based on GPR and XGBoost are trained for each of the three CO_2_ injection rates. This approach permits the models to best quantify any rate-specific behaviors within the input-output relationship as possible. During GPR training, kernel hyperparameters are optimised by maximizing the log marginal likelihood^73,74^. For XGBoost, model is trained iteratively, with early stopping employed based on performance on the test set (used as a validation set for early stopping purposes) to determine the optimal number of boosting rounds for the models after the training occurred^61^.

A number of standard statistical metrics are used to measure the predictive performance of the trained models on the unseen test sets. These include:

- R-squared (R²): The coefficient of determination or the proportion of variance of the target variable that can be predicted from the input feature. Values closer to 1 indicate a better fit.

7 $$\:{\mathrm{R}}^{2}=1-\frac{{\sum\:}_{\mathrm{i}=1}^{{\mathrm{N}}_{\mathrm{t}\mathrm{e}\mathrm{s}\mathrm{t}}}{\left({\mathrm{y}}_{\mathrm{i},\mathrm{t}\mathrm{r}\mathrm{u}\mathrm{e}}-{\mathrm{y}}_{\mathrm{i},\mathrm{p}\mathrm{r}\mathrm{e}\mathrm{d}}\right)}^{2}}{{\sum\:}_{\mathrm{i}=1}^{{\mathrm{N}}_{\mathrm{t}\mathrm{e}\mathrm{s}\mathrm{t}}}{\left({\mathrm{y}}_{\mathrm{i},\mathrm{t}\mathrm{r}\mathrm{u}\mathrm{e}}-\stackrel{-}{{\mathrm{y}}_{\mathrm{t}\mathrm{r}\mathrm{u}\mathrm{e}}}\right)}^{2}}$$

- Mean Absolute Error (MAE): The average of absolute differences between predicted values and actual values which gives a measure of average error magnitude.

8 $$\:\mathrm{M}\mathrm{A}\mathrm{E}=\frac{1}{{\mathrm{N}}_{\mathrm{t}\mathrm{e}\mathrm{s}\mathrm{t}}}{\sum\:}_{\mathrm{i}=1}^{{\mathrm{N}}_{\mathrm{t}\mathrm{e}\mathrm{s}\mathrm{t}}}\left|{\mathrm{y}}_{\mathrm{i},\mathrm{t}\mathrm{r}\mathrm{u}\mathrm{e}}-{\mathrm{y}}_{\mathrm{i},\mathrm{p}\mathrm{r}\mathrm{e}\mathrm{d}}\right|$$

- Root Mean Squared Error (RMSE): The square root of the average of squared differences.

9 $$\:\mathrm{R}\mathrm{M}\mathrm{S}\mathrm{E}=\sqrt{\frac{1}{{\mathrm{N}}_{\mathrm{t}\mathrm{e}\mathrm{s}\mathrm{t}}}{\sum\:}_{\mathrm{i}=1}^{{\mathrm{N}}_{\mathrm{t}\mathrm{e}\mathrm{s}\mathrm{t}}}{\left({\mathrm{y}}_{\mathrm{i},\mathrm{t}\mathrm{r}\mathrm{u}\mathrm{e}}-{\mathrm{y}}_{\mathrm{i},\mathrm{p}\mathrm{r}\mathrm{e}\mathrm{d}}\right)}^{2}}$$

In Eqs. (7–9), y_i, true_ is the actual value from the test set, y_i, pred_ is the predicted value using the ML models and $\:{\overline{\mathrm{y}}}_{\mathrm{true}}\:$is equal to the mean of the actual values in the test set. All these computations have been carried out using python with scikit-learn and XGBoost libraries.

## ML-based characterisation of CO_2_ geosequestration

**Fig. 10:**
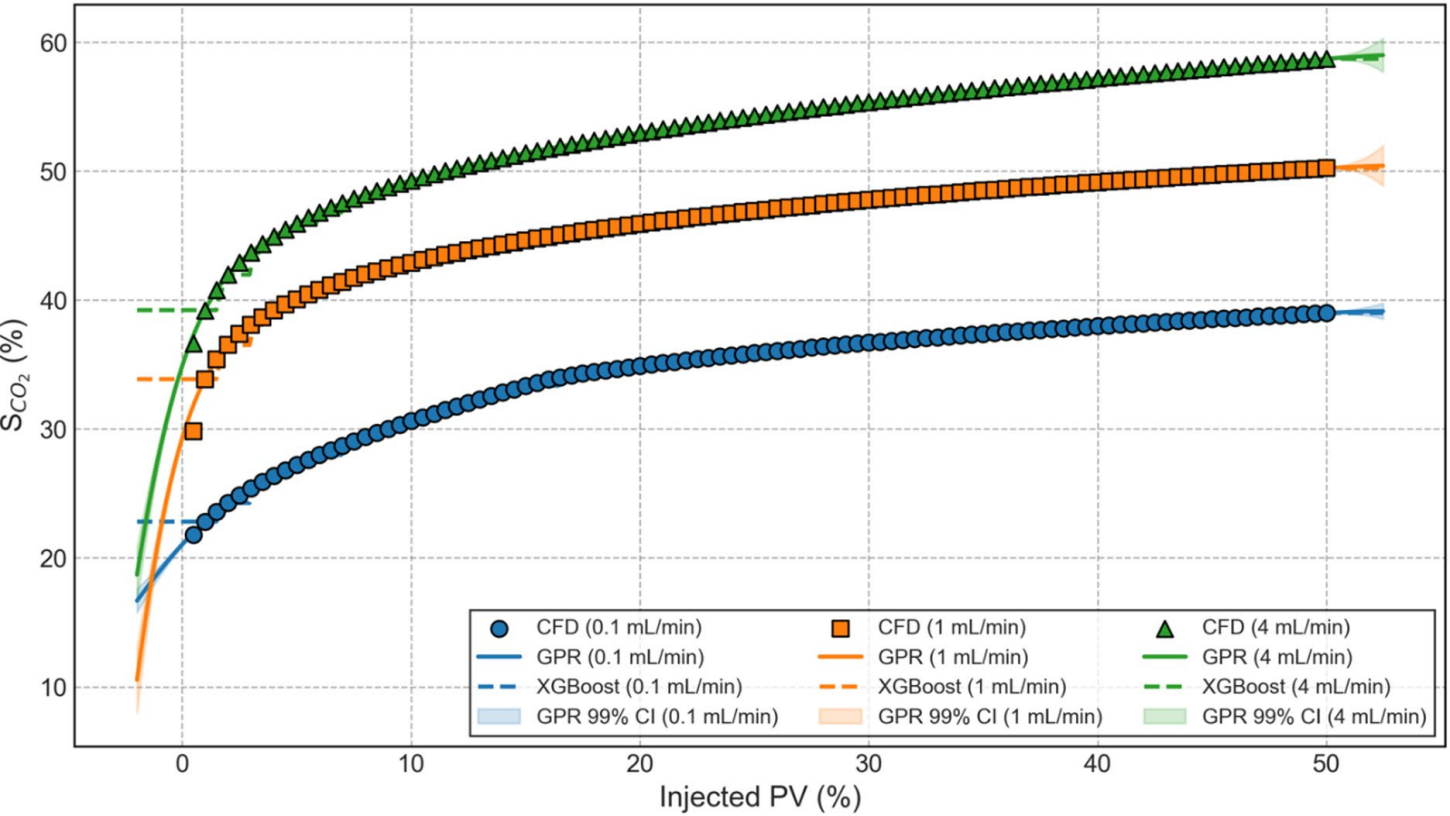
Validation of GPR and XGBoost data against the actual S_CO2_ values.

Predictive performance of trained Gaussian Process Regression (GPR) and Extreme Gradient Boosting (XGBoost) models is evaluated and compared in order to characterise CO_2_ geosequestration with the aim to establish whether probabilistic or deterministic ML models perform superior. Well validated CFD data is used to build both these models, which also serves to validate these models. Figure 10 depicts the data of S_CO2_ versus injected PV (same as Fig. 7) along with the results of trained GPR and XGBoost ML models at the three different injection rates considered in this study. CFD data is represented by markers, GPR data with solid lines and XGBoost data with dotted lines. As GPR is a probabilistic model, its 99% confidence band is shown in the highlighted areas. It is quite clear from the figure that both GPR and XGBoost predicted S_CO2_ matches very closely with the CFD/actual data for all the injection rates. Upon careful inspection, it can be noticed that at very low injected PV values, XGBoost predicted data deviates away from the actual data. A potential reason for this could be the limited training dataset available, which is ideal for GPR but not for XGBoost however, XGBoost is significantly faster than GPR. Thus, there is a need to further evaluate both these ML models and carry out a robust comparative analysis.

**Fig. 11:**
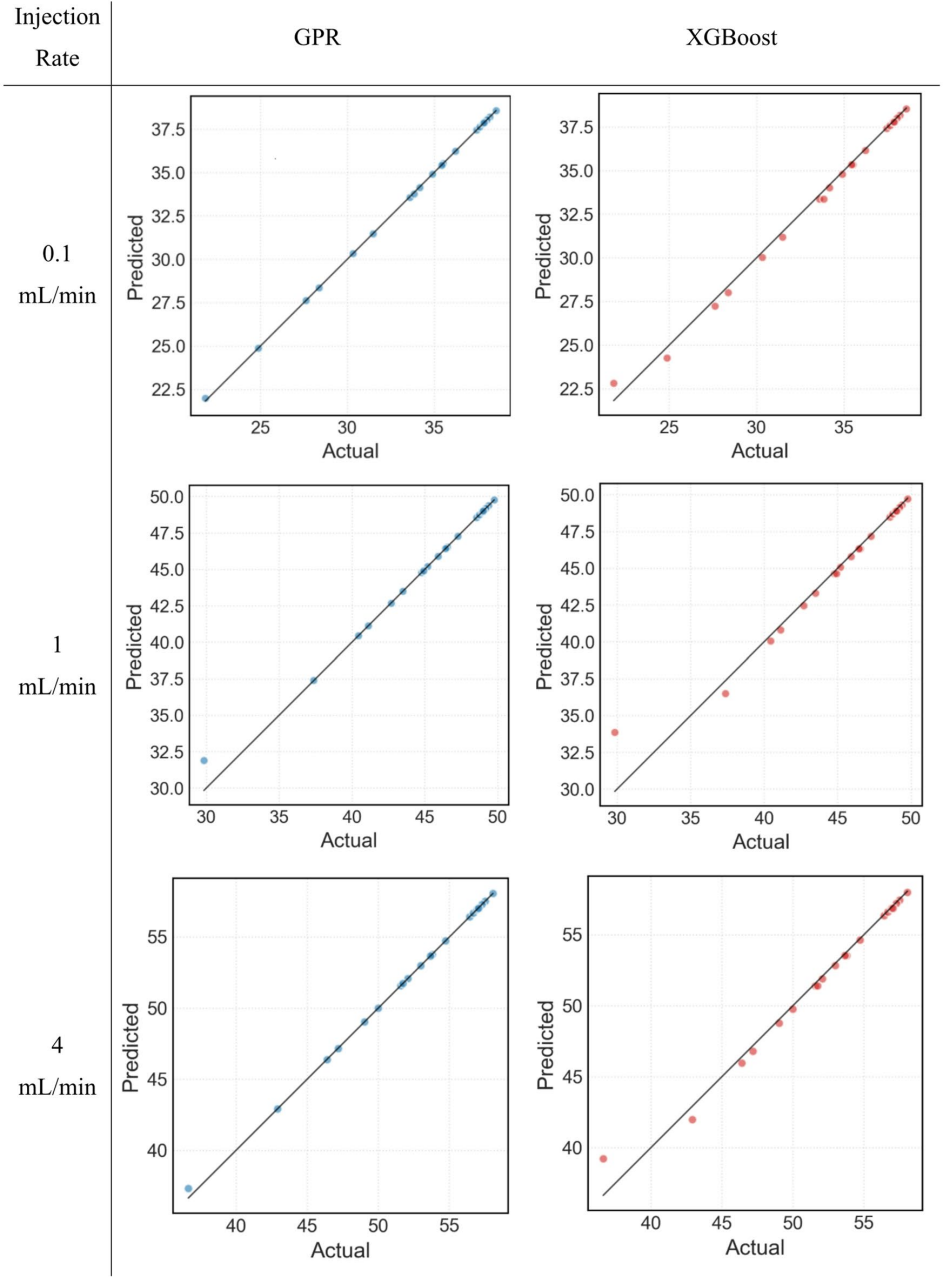
Scatter plots of predicted versus actual values of S_CO2_ at different injection rates using GPR and XGBoost.

Scatter plots of actual versus predicted S_CO2_ from GPR and XGBoost for the different injection rates are shown in Fig. 11. In case of GPR, almost all data points except for the lowest S_CO2_ in case of intermediate injection rate lie on the line of equality, clearly demonstrating that GPR predicts CO_2_ saturation accurately. In case of XGBoost, lowest two S_CO2_ values do not lie on the line of equality but the rest of the data does, showing that XGBoost predicts CO_2_ saturation accurately but not at lower injected PV values i.e. in the initial stages of CO_2_ injection into the reservoir. The results presented in Fig. 11 are in-line with the results of Fig. 10, suggesting that GPR might be a better option for characterising CO_2_ geosequestration in naturally fractured reservoirs however, not conclusively.

**Fig. 12:**
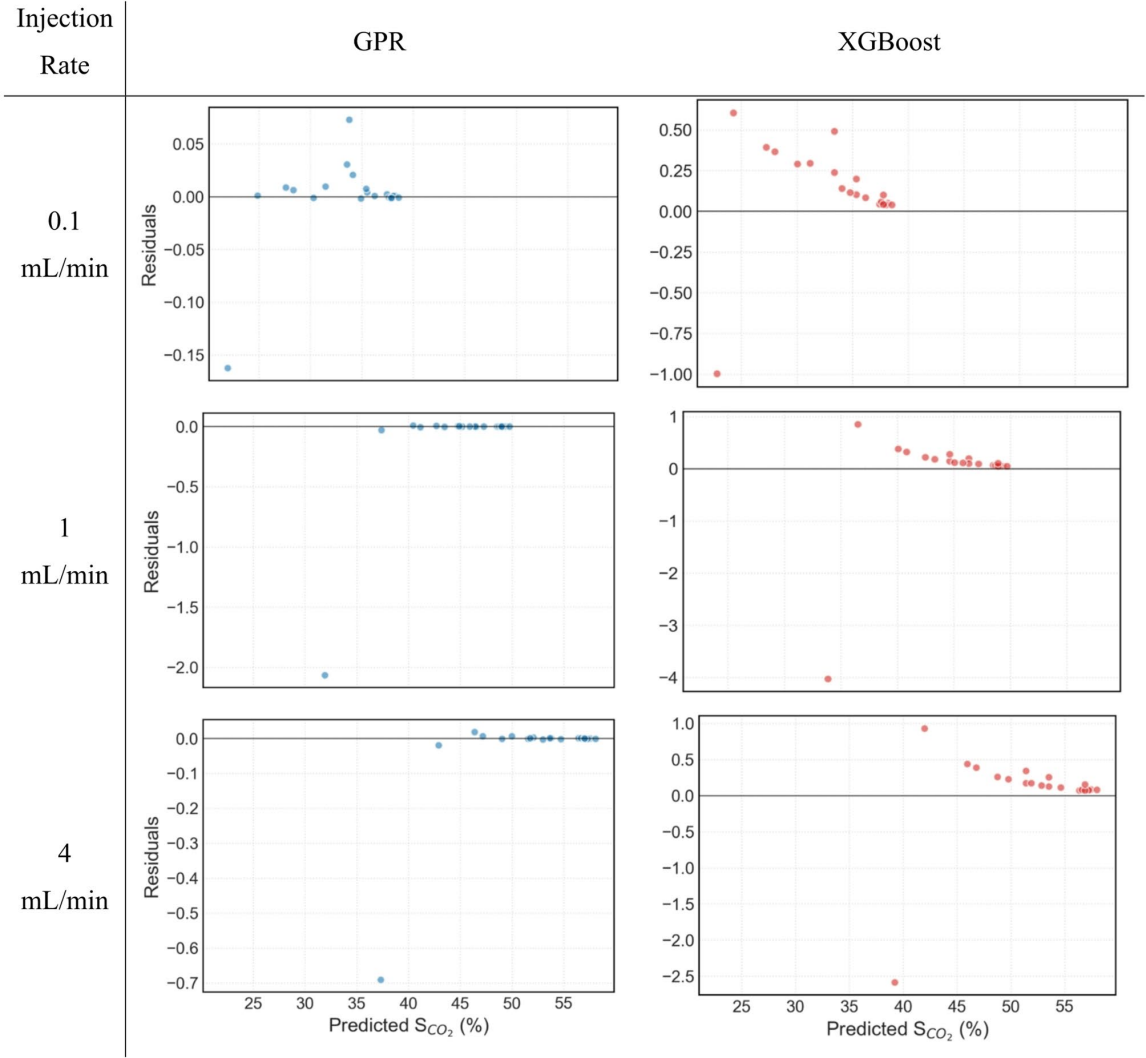
Residuals of predictive performance of GPR and XGBoost for different injection rates.

Figure 12 depicts the residual plots of S_CO2_ prediction from GPR and XGBoost at different injection rates, where residual value simply means the actual value minus the predicted value. Residual plots help identify any bias or misspecification in the prediction modelling and thus are a powerful tool for evaluating ML models. Examining the plots one can clearly see that the residual values for the GPR model are significantly smaller than for XGBoost indicating a tighter fit around zero value. In all the residual plots shown, one data point (left-most) is an outlier. Ignoring this outlier, all data points for intermediate and high injection rates in GPR model are either on the zero residual line, or are evenly distributed across it, indicating fairly random distribution and thus excellent fit. For low injection rate of GPR, there seems to be a bias towards overpredicting S_CO2_ however there doesn’t seem to be any trend observed in this overprediction rather this overprediction is observed only in the region of S_CO2_ 30% − 35%. Overall, GPR residual show excellent fit of the predicted S_CO2_ with the actual S_CO2_.

In case of XGBoost residual plots, ignoring the left-most outlier, one can clearly see that the magnitude of spread is considerably higher than observed in case of GPR. Moreover, XGBoost is shown here to always overpredict S_CO2_ indicating bias in predictive capabilities. Further analysing XGBoost residual plots it can be noticed that as S_CO2_ value increases, which also means increasing injected PV values, the residuals decrease and try to converge on the zero residual line. This trend clearly demonstrates systematic error in data prediction in the GPR model. The residual plots thus explicitly indicate that GPR is more stable and accurate in predicting CO_2_ geosequestration in naturally fractured reservoirs, while XGBoost fails to accurately predict the nonlinear response of the reservoir at all injection rates considered, highlighting its limitations.

**Fig. 13:**
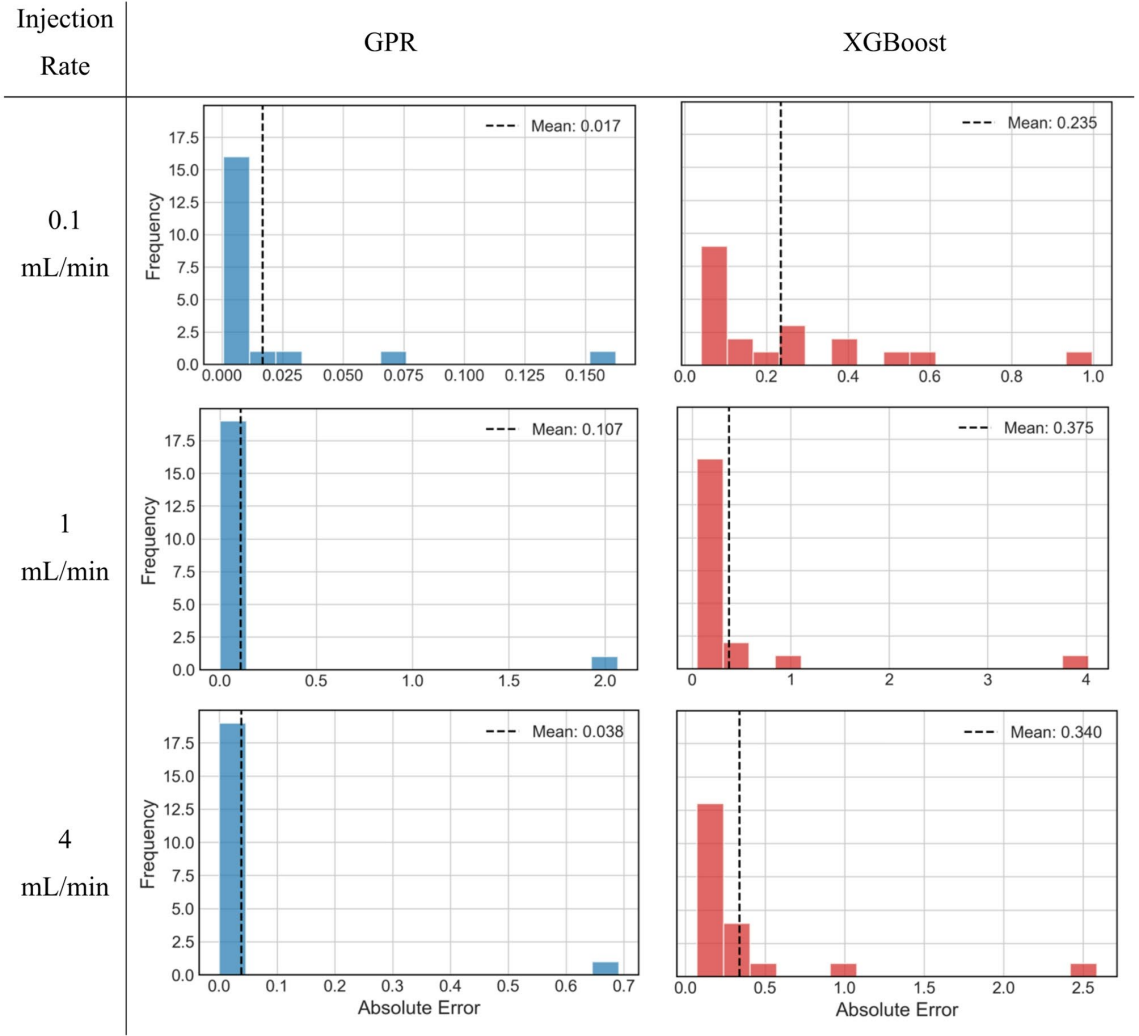
Absolute error histograms of GPR and XGBoost at different injection rates.

Figure 13 shows histograms of absolute error from both GPR and XGBoost at different injection rates considered. The absolute error is calculated using Eq. (8). Histograms depict the spread of error in predictive performance of ML models. The outlier observed previously, and shown here as well (highest error; right-most), if ignored than it is clearly seen that the absolute error of GPR model is very close to zero, especially in case of intermediate and high injection rates. The mean absolute error, also shown in the figure as vertical dotted line, for low to high injection rates of GPR model are 0.017, 0.107 and 0.038 respectively. These values are quite small indicating very high precision and accuracy. In case of XGBoost model, one can see a wider spread of absolute error, while the mean values for low to high injection rates are 0.235, 0.375 and 0.34 respectively. Thus, absolute error from XGBoost in predicting S_CO2_ at low injection rate is 12.8 times higher than absolute error from GPR. At intermediate and higher injection rates, this difference is 2.5 times and 7.9 times, clearly indicating that GPR is significantly more accurate in predicting CO_2_ geosequestration than XGBoost, albeit its faster modelling and predictive capabilities.

**Fig. 14:**
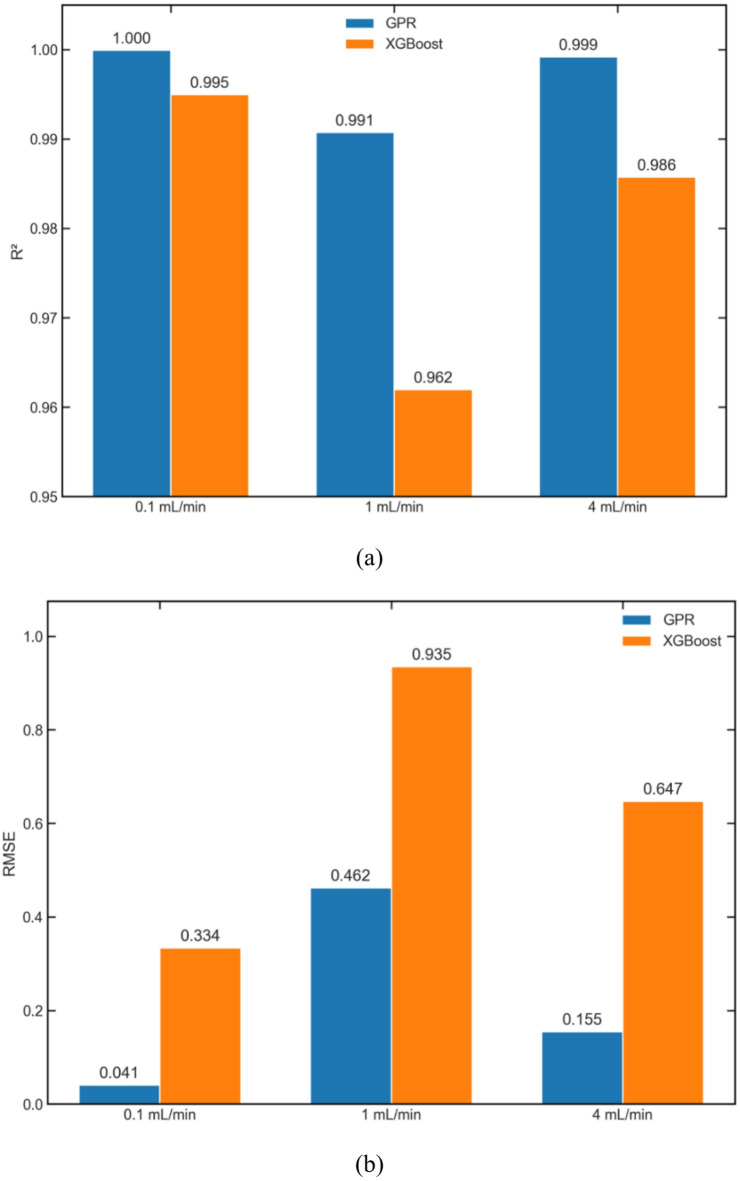
Statistical comparison of GPR and XGBoost at different injection rates using (**a**) R^2^ and (**b**) RMSE variations.

In order to firmly ascertain that GPR is superior to XGBoost in characterising CO_2_ geosequestration in fractured rocks, standard statistical metrices of Eqs. (7) and (9) i.e. R^2^ and RMSE, from both these modes are compared in Fig. 14. It can be seen in Fig. 14(a) that R^2^ values from GPR are higher than from XGBoost. The lowest R^2^ value from GPR is 99.1% at intermediate injection rate, while from XGBoost is 96.2% at the same injection rate. This indicates that the predictive accuracy of GPR is superior to XGBoost. Further analysing RMSE in Fig. 14(b), it can be seen that RMSE values from GPR are significantly lower than from XGBoost as RMSE is more sensitive to outliers. That’s the reason why RMSE values shown in Fig. 14 are higher than MAE values in Fig. 13. RMSE values from XGBoost at low to high injection rates are 7.1 times, 102% and 3.1 times higher than from GPR respectively, indicating higher error. Moreover, XGBoost’s performance degrades at higher injection rates. Thus, GPR is more accurate and robust in prediction CO_2_ geosequestration in naturally fractured reservoirs, and is concluded here as the preferred model for reservoir characterisation.

## Advanced ML model with expanded input features

The initial analysis in Section “ML-based characterisation of CO_2_ geosequestration” demonstrated the high potential of ML surrogates, particularly GPR, using injected PV as the primary predictive feature for individual injection rates. In order to create a more robust and generalised surrogate model, addressing the limitations of small dataset and overfitting, a comprehensive ML-based model is developed, trained and tested. This advanced model is based on a consolidated dataset from 27 unique scenarios (CFD cases), incorporating critical reservoir properties as input features. The goal is to test the advanced model’s ability to generalise and make predictions for entirely unseen CO_2_ injection cases.

The dataset for the advanced ML model consists of 2700 data points, derived from 27 CFD runs. A key difference in this model is the expansion of the input feature dataset, from a single parameter to five parameters. These parameters include porosity (φ), matrix permeability (k_matrix_$\:)$, fracture aperture (h), entry capillary pressure ($\:{{\mathrm{P}}^{\mathrm{l}}}_{\mathrm{e}\mathrm{c},{\upbeta\:}}$) and the original input feature i.e. injected PV. The output target remains the same i.e. CO_2_ saturation (S_CO2_).

To rigorously test for generalisability and prevent any data leakage or overfitting, a held-out case validation is carried out. Instead of a simple random split, the dataset was split by Case ID:

- Training Set: 21 unique cases (2100 data points).
- Test Set: 6 unseen cases (600 data points).

The goal is to test the model’s predictive capability for those reservoir conditions that is unseen to the model.

A systematic hyperparameter search was conducted to optimise the XGBoost model. A GridSearchCV (Grid Search Cross-Validation) with 5 folds was employed, testing 108 candidate combinations to find the set of parameters that minimised the cross-validated error. The rationale for this strategy is to ensure the model’s robustness and prevent manual tuning bias. The search space and the resulting optimal parameters are summarised in Table 2.

**Table 2:** XGBoost hyperparameter optimisation via 5-fold GridSearchCV.

Parameter	Description	Search Space	Best Value Found
n_estimators	Number of decision trees	[100, 200, 300]	300
max_depth	Maximum depth of a tree	[3, 4, 5]	3
learning_rate	Step size shrinkage	[0.05, 0.1, 0.2]	0.2
subsample	Fraction of samples used for fitting trees	[0.8, 1.0]	1.0
colsample_bytree	Fraction of features used for fitting trees	[0.8, 1.0]	1.0

For the GPR model, the kernel hyperparameters were optimised during the training process by maximising the log-marginal-likelihood. The final learned kernel is presented in Table 3, summarising the five different length scales corresponding to the five input features, which confirms that the model is treating each feature’s influence differently.

**Table 3:** Optimised GPR kernel.

Kernel Component	Parameter	Value
ConstantKernel	constant_value	0.641**2 (0.411)
RBF	length_scale (for Porosity)	3.37
RBF	length_scale (for Permeability)	8.71
RBF	length_scale (for Aperture)	1.24
RBF	length_scale (for Entry Pressure)	5.55
RBF	length_scale (for PV)	0.146
WhiteKernel	noise_level	2.63 × 10^5^

To provide a direct comparison with the probabilistic GPR model, uncertainty quantification of XGBoost model is carried out. This is achieved using a bootstrapping ensemble, where 20 separate XGBoost models are trained on different resampled (bootstrapped) subsets of the training data. The final prediction is the mean of these 20 models, and the 99% confidence interval is calculated from the 0.5th and 99.5th percentiles of their collective predictions.

The models are trained on 21 cases and are evaluated on 6 held-out cases. The performance metrics, shown in Table 4, clearly demonstrate the highly accurate predictive capabilities of the models. The GPR model achieved an R² of 0.9967 and an RMSE of 0.005, while the optimised XGBoost model achieved an R² of 0.9909 and an RMSE of 0.008. This clearly demonstrates that both the models, when provided with sufficient data and features, can generalise new scenarios accurately.

**Table 4:** Advanced model’s performance on held-out test set.

Model	*R*²	MAE	RMSE
GPR	0.9967	0.002	0.005
XGBoost	0.9909	0.004	0.008

**Fig. 15:**
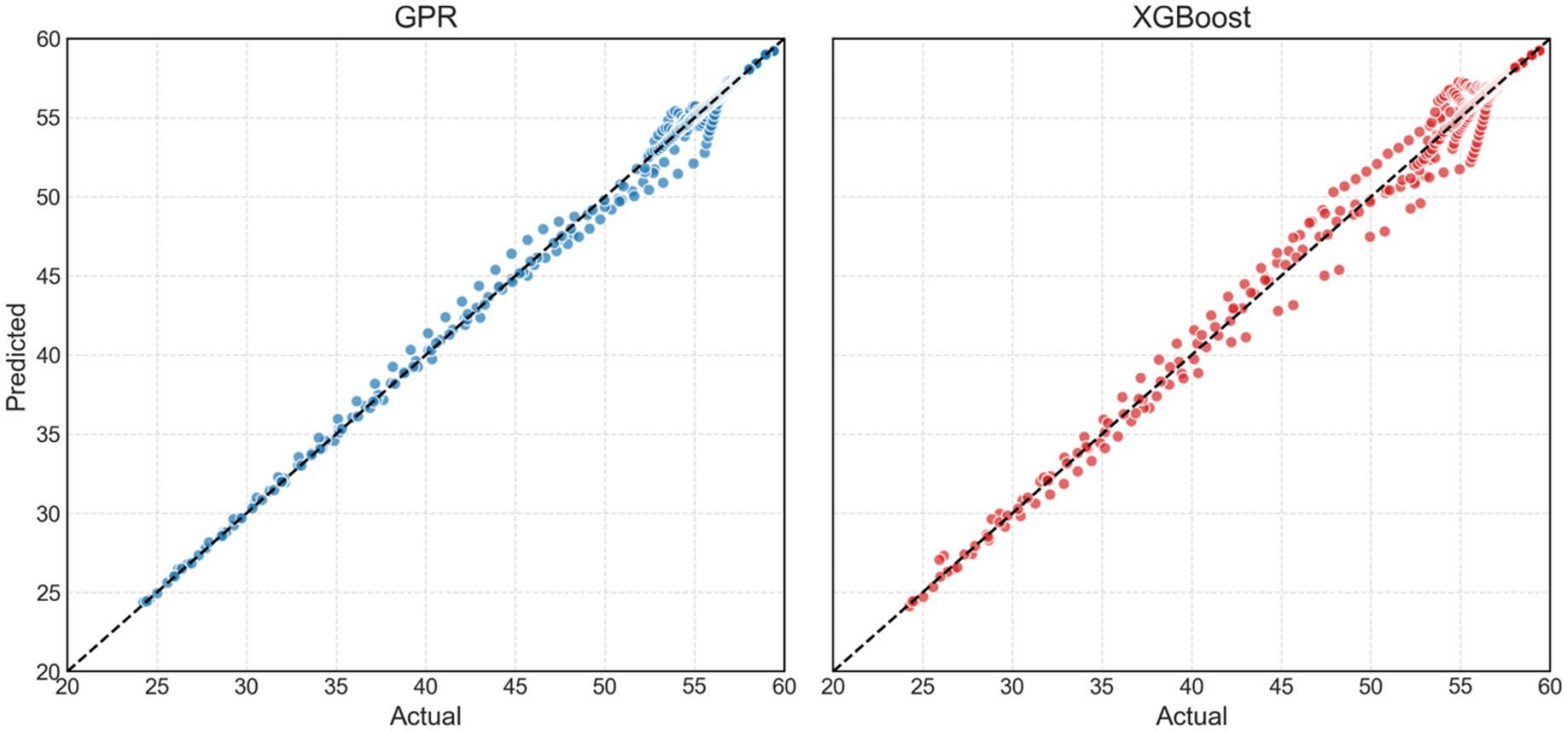
Scatter plots comparing predicted and actual S_CO2_ values for the GPR and XGBoost models on the held-out test set.

Figure 15 further depicts the predictive performance of both these models. The scatter plot compares the model’s predicted S_CO2_ values with the actual S_CO2_ values from the 6 unseen CFD cases. It can be seen that for both GPR and XGBoost models, the 600 data points form an a tight cluster on the 1:1 line (dashed black). This confirms that the models are not systematically over- or under-predicting and that the error (RMSE) is very low.

**Fig. 16:**
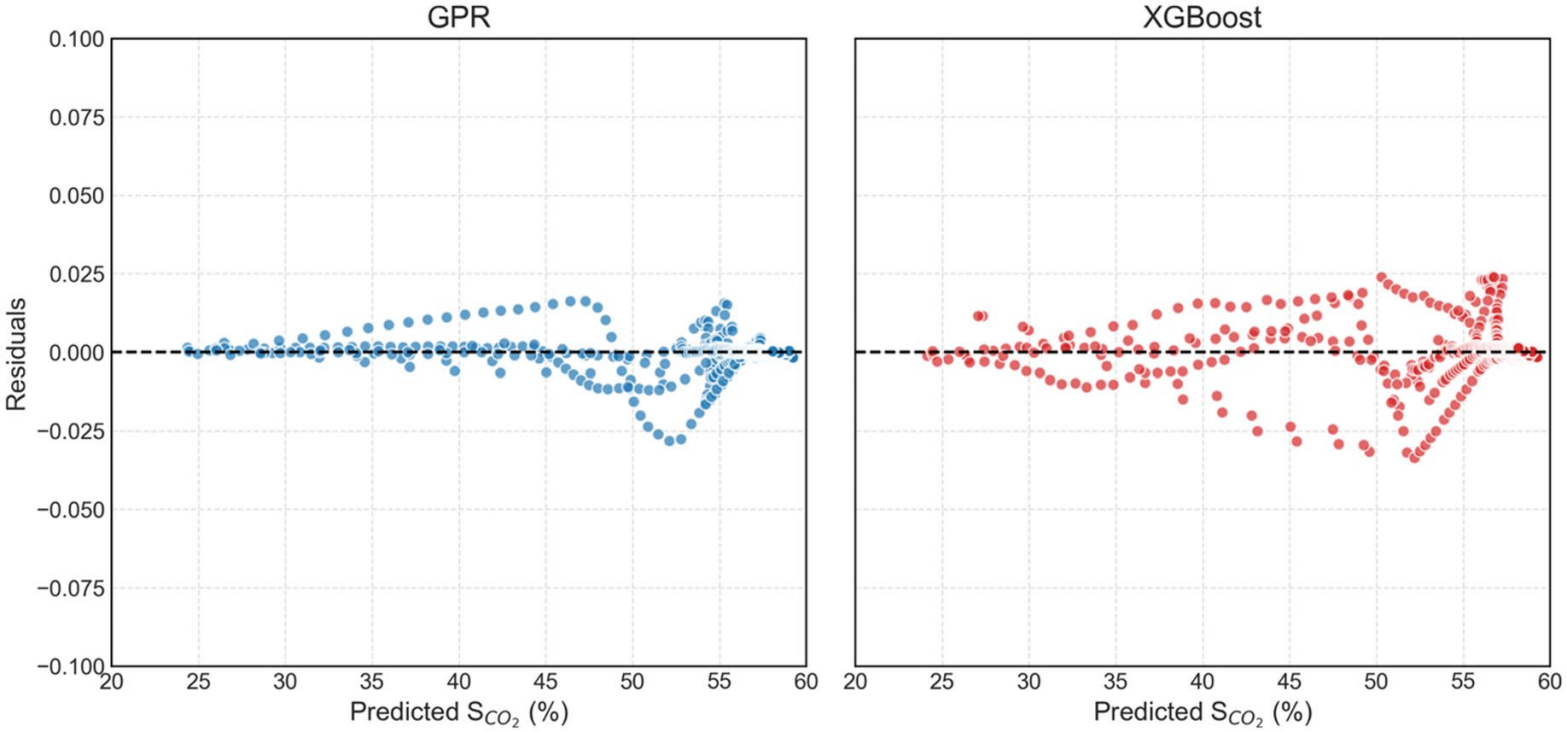
Residual plots of predictive performance of GPR and XGBoost models on the held-out test set.

Further analysing the predictive performance of both GPR and XGBoost models on the held-out dataset, Fig. 16 depicts the residual plots from both the models. As seen earlier in Fig. 12, the data points are scattered tightly and randomly around the zero residual line with no clear bias. However, comparing GPR scatter plot with XGBoost scatter plot, it can be seen that the scatter of data points around the zero residual line is higher in case of XGBoost model. Thus, the results shown in Fig. 16 reaffirm the results obtained in Fig. 12.

**Fig. 17:**
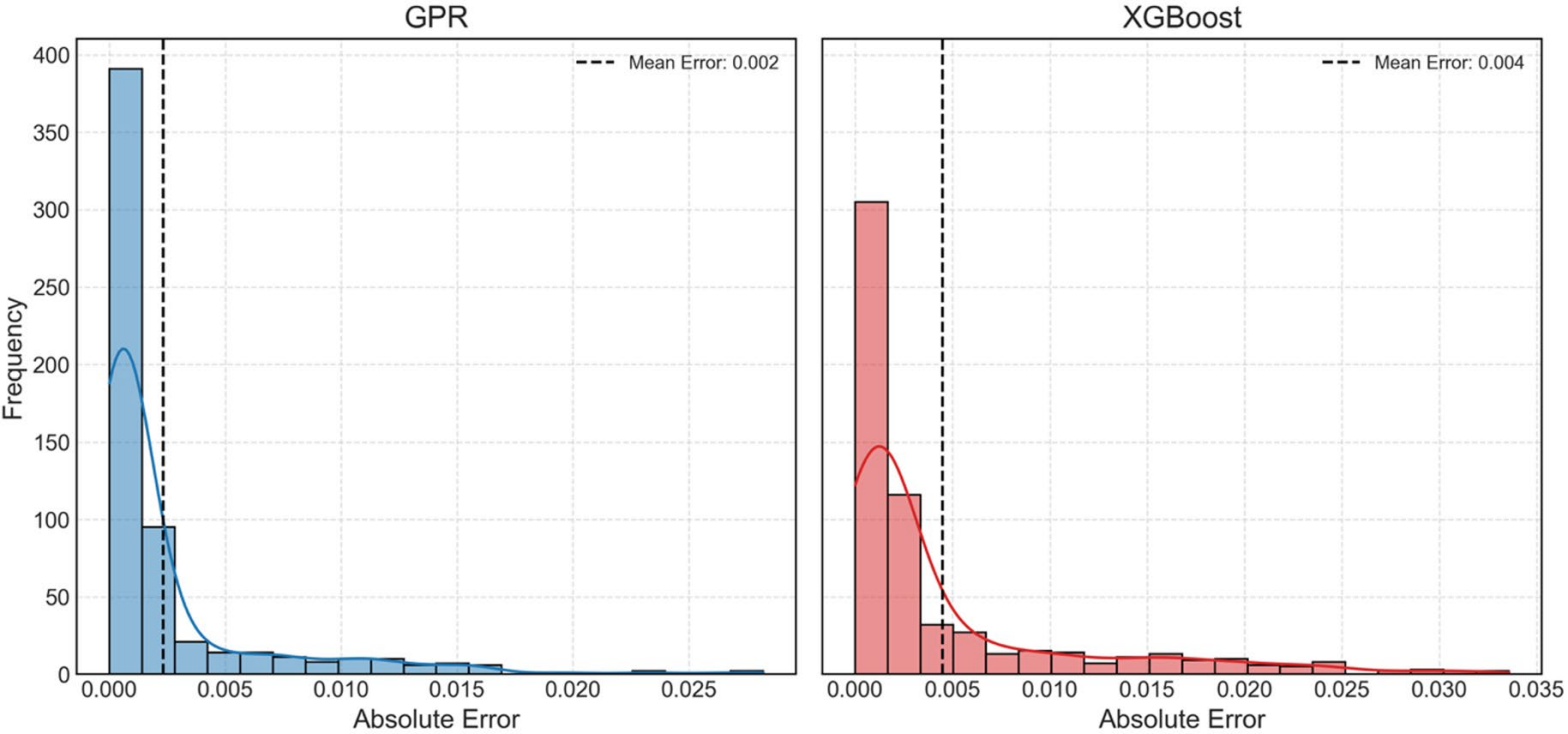
Absolute error histograms of GPR and XGBoost on the held-out test set.

Figure 17 shows the histogram of absolute error for both the GPR and XGBoost models on the held-out dataset. It can be seen that the absolute error of GPR model is closer to zero compared to XGBoost model. The mean absolute error for GPR is 0.002, while for XGBoost, it is 0.004. These values are very small, indicating high precision and accuracy of both the models.

**Fig. 18:**
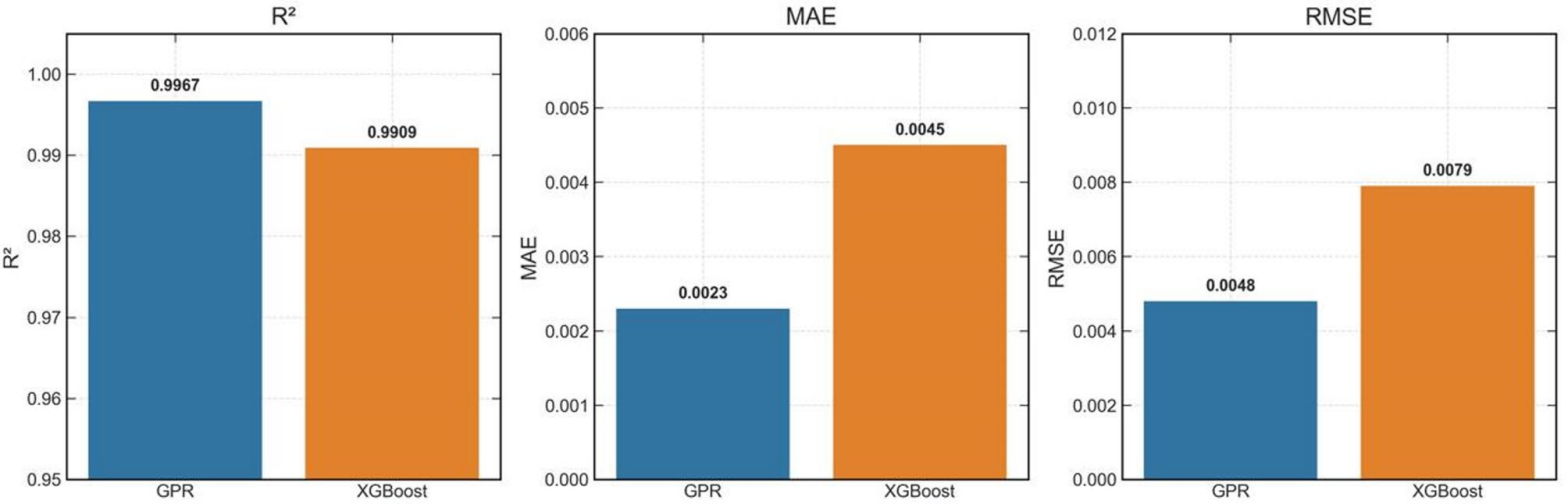
Statistical comparison of GPR and XGBoost performance on the held-out test set.

The standard statistical metrices of both the models are compared and shown in Fig. 18. The first figure (left) compares the R^2^ values, the second one (middle) compares the MAE values and the third one (right) compares the RMSE values of both the models. It can be clearly seen that R^2^ of both the models is very high, while MAE and RMSE are very low. For GPR, the R^2^, MAE and RMSE are 99.67%, 0.23% and 0.48% respectively. Similarly, for XGBoost model, these values are 99.09%, 0.45% and 0.79% respectively. Thus, GPR outperforms XGBoost in predictive performance related to CO_2_ geosequestration.

**Fig. 19:**
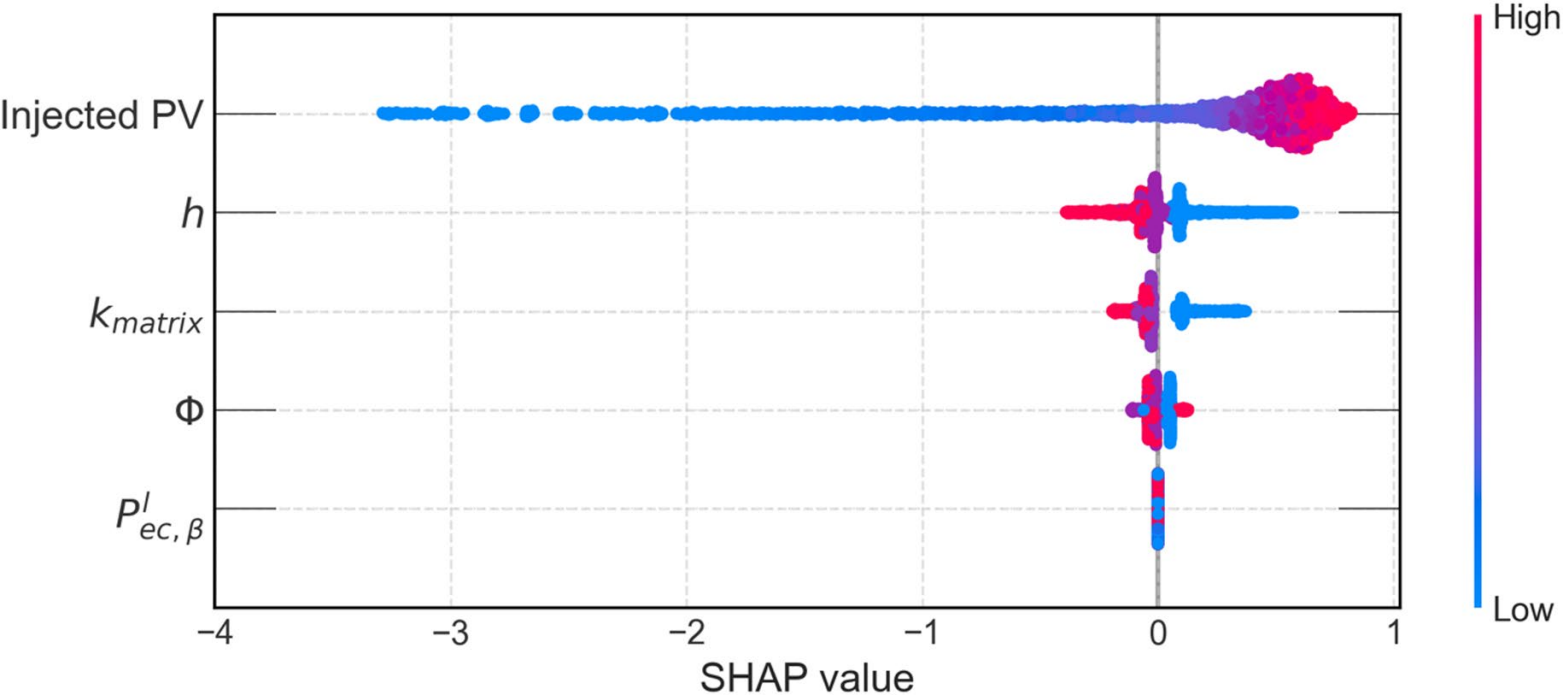
SHAP summary plot of the input features for the XGBoost model.

Input feature importance analysis is carried out using SHAP (SHapley Additive exPlanations) analysis, for the trained XGBoost model, and is shown in Fig. 19. SHAP ranks the five input features by their importance (top-to-bottom) and shows the impact of each feature on CO_2_ geosequestraion (through S_CO2_). Each point in the figure represents a single prediction. As expected, injected PV is the most important feature as it drives the dynamic sequestration process. The static reservoir properties also have impact on CO_2_ geosequestration with fracture aperture (h) being the second most influential input feature after injected PV. The high values of h (red dots) tend to increase or decrease S_CO2_ depending on the injection strategy. Porosity (φ) and matrix permeability (k_matrix_) have relatively lower SHAP values, indicating secondary influence on S_CO2_. This is true as both these parameters dictate CO2 transfer from fracture into matrix, and vice-versa, rather than controlling CO_2_ front propagation. Entry capillary pressure $\:{{\mathrm{P}}^{\mathrm{l}}}_{\mathrm{e}\mathrm{c},{\upbeta\:}}\:$ predominantly has slightly negative SHAP values, indicating stronger resistance to CO_2_ propagation into the fractured reservoir as entry capillary pressure increases.

**Fig. 20:**
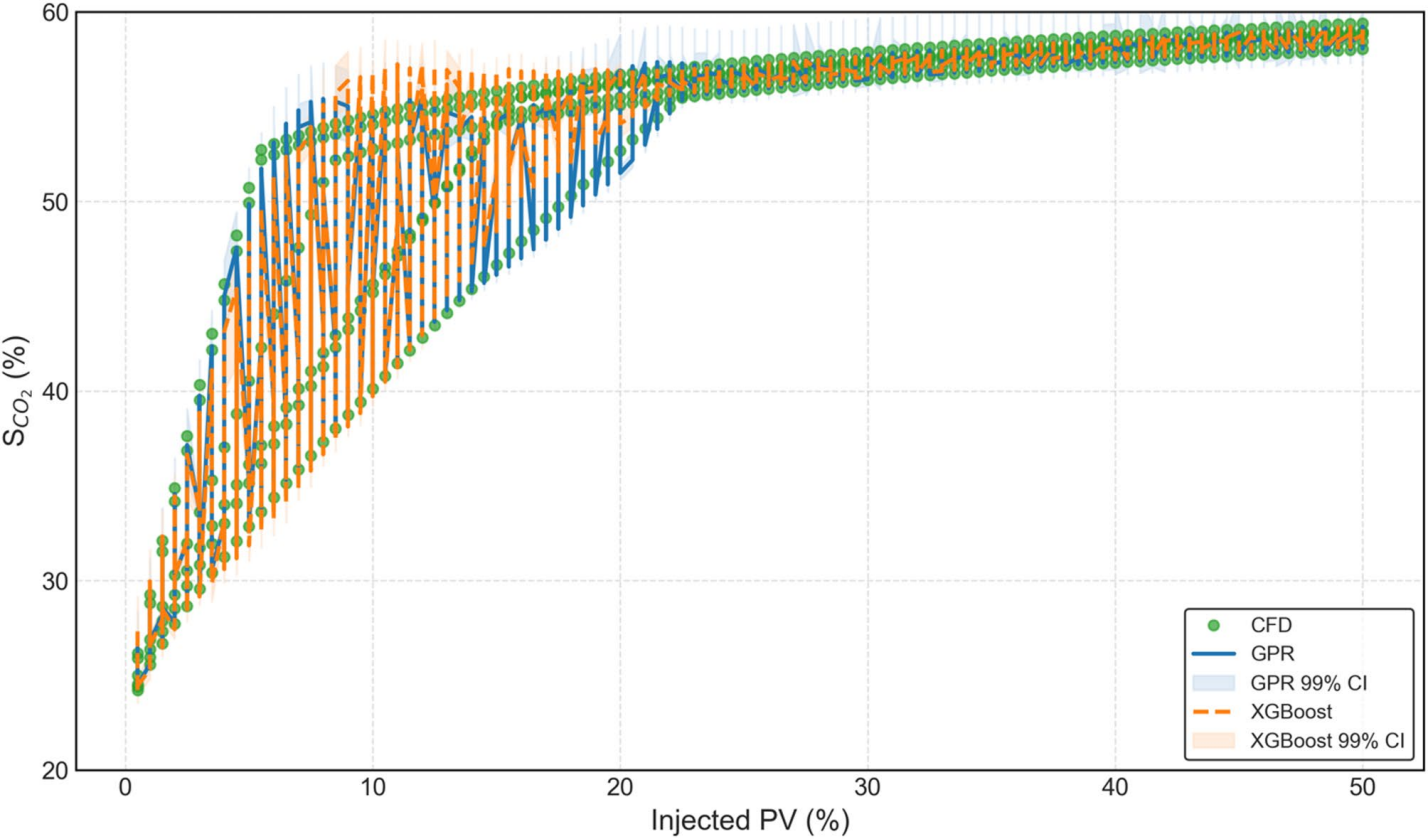
Comparison of GPR and XGBoost model predictions against actual CFD data for the held-out test set.

Figure 20 compares the model predictions, including uncertainty, against the actual 600 CFD-based test data points of S_CO2_. The GPR (blue) and XGBoost (orange) predictions are overlaid, along with their respective 99% confidence intervals (shaded regions). It can be seen that that the actual S_CO2_ data points fall almost entirely within the 99% confidence intervals of both the models. This clearly demonstrates that the advanced models are quite accurate in predicting S_CO2_.

**Fig. 21:**
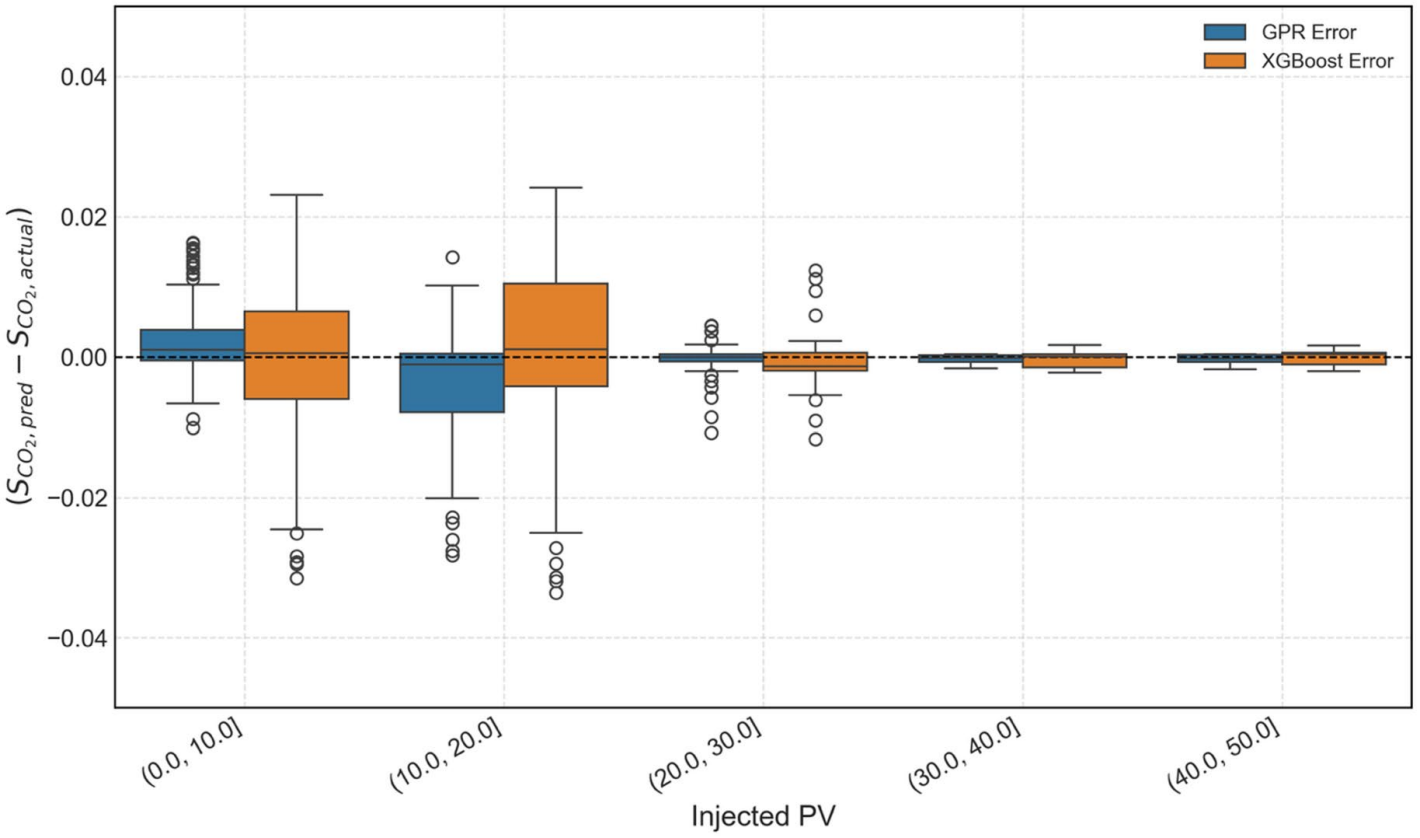
Boxplot analysis of prediction error for the GPR and XGBoost models, binned by Injected PV (%) intervals for the held-out test set.

Figure 21 depicts boxplot of the prediction error (predicted – actual) binned by the injected PV. The boxes show the median and interquartile range of the errors. The plot demonstrates that the prediction error for both the models is consistently low and centred around zero across all stages of injection, especially at higher injected PV values. The median error (centre line) for all bins is almost zero, and the boxes (error distribution) are compact. This is a significant finding; while the baseline model developed earlier showed XGBoost struggling at low injected PV values, the advanced multi-feature model is more accurate and stable even at early injection stage.

## Practical implications of ML for CO_2_-EOR

The successful development and implementation of a ML surrogate model for accurately predicting S_CO2_ ​​​​ holds considerable practical implications for advancing CO_2_-EOR projects and CO_2_ geosequestration^75,76^. Alongside the computational efficiency and predictive accuracy of the ML model developed, rapid scenario analysis and optimisation is one of the most important practical aspects of ML in reservoir performance prediction. The ML surrogates with almost real-time prediction times enable rapid exploration of a much wider range of operational parameters like fine-tuning injection schedules beyond the three injection rates tested^77^. This accelerates sensitivity analyses and facilitates more comprehensive optimisation of injection strategies to maximise CO_2_ geosequestration and EOR simultaneously, potentially leading to more energy-efficient operations^77^. Table 5 provides a summary of the computational costs involved with both CFD and ML as a comparison to highlight ML’s almost real-time prediction capabilities. It can be seen that where a single 2D CFD run takes around 6 h which includes a single fracture in a porous medium, the ML model training and testing requires less than a minute. This reduction of computational time by almost 3 orders of magnitude clearly demonstrates the potential of ML surrogate modelling for real-time analysis and optimisaiton of CO_2_ injection.

**Table 5:** Computational cost comparison between CFD and ML.

Component	Task	Platform	Runtime
CFD (baseline modelling)	2D pore-scale single fracture modelling	8 core CPU; 16 GB RAM	~ 6 h
CFD (parametrisation)	27 runs	8 core CPU; 16 GB RAM	~ 7 days
ML (training & testing)	270 samples	Laptop	< 60 s
ML (predictions)	1000 scenarios	Laptop	< 5 s

**Fig. 22:**
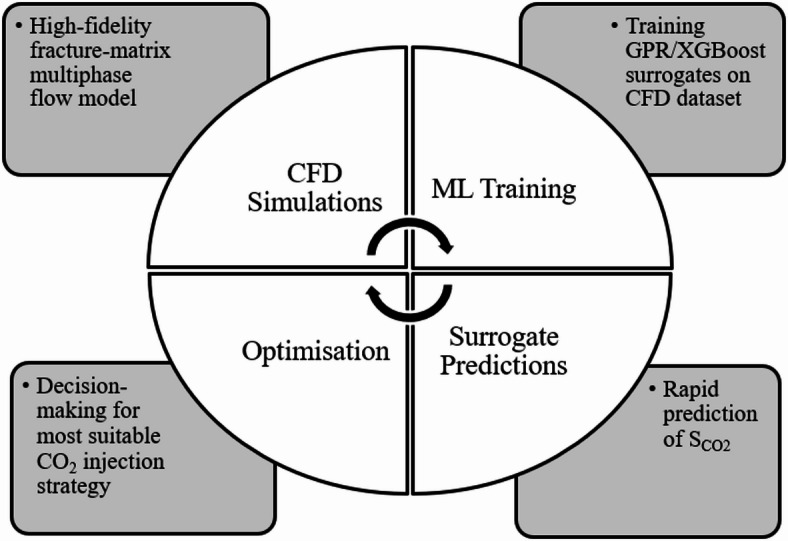
CFD-ML closed-loop optimisation workflow.

Enhanced uncertainty quantification is another important practical implication of ML surrogates. The GPR model particularly incorporates inherent quantifications of uncertainties (confidence intervals) into predictions. This allows for improved uncertainty propagation from inputs like reservoir properties, to prediction outputs, which results in more robust risk estimation and decision-making for CO_2_-EOR projects that are crucial for long-term carbon storage security^76^. ML also provide support for field development planning as conventional field development often relies on numerous high-fidelity and computationally expensive simulations. Fast ML surrogates can be integrated into larger workflows, such as history matching or production forecasting, significantly reducing the computational burden without substantial loss of accuracy for the parameters modelled^76,78^. Demonstrating the practical implementation of ML surrogates in a closed-loop decision-making/optimisation workflow for CO_2_ injection, Fig. 22 provides an illustrative overview of how the current work will be implemented in real-life CO_2_ underground storage projects.

Real-time and precise prediction of S_CO2_ under different conditions allows improved estimation of the dynamic storage capacity of the reservoir as well as the effectiveness of various injection schemes in terms of achieving long-term and secure underground carbon storage, thus contributing to a reduced net emissions profile This not only aids in better understanding of complexities involved in CO_2_ geosequestration but also helps in bridging the gaps in complex multi-flow physics with practical applications of ML, enabling sophisticated predictive capabilities more accessible for routine engineering tasks. By providing tools for faster and more extensive analysis, ML models directly contribute towards improving the efficiency of hydrocarbons extraction from unconventional reservoirs while enhancing the environmental co-benefit of carbon capture and storage, thereby supporting sustainable energy objectives and strategies for minimising the carbon footprint of energy production for a more sustainable future^76,77^.

## Conclusions

The study presents a novel approach towards characterising CO_2_-EOR through the development of a hybrid CFD-ML approach for naturally fractured reservoirs where CFD generated data of CO_2_ saturation is used to train and test ML models. Utilising discrete fracture modelling approach, CFD results for low-to-high CO_2_ injection rates and at incremental injected PV values are analysed in detail to evaluate the dynamic propagation of CO_2_ front in the rock sample. This is carried out using difference contours of CO_2_ saturation which has clearly revealed the advantages and disadvantages of different CO_2_ injection rates. Low injection rates although delays breakthrough, they lead to lower sweep efficiency, front displacement and thus, lower CO_2_ storage in the reservoir due to domination of capillary forces. Meanwhile, high injection rates show higher CO_2_ storage and effective oil RF due to dominance of viscous forces but at the same time it is an impractical injection strategy due to exorbitant entry pressure requirements. Thus, intermediate injection rates are shown to be the optimal solution to this where a balance between capillary and viscous forces is seen, leading to high CO_2_ saturation in the reservoir at moderate entry pressures, enhancing CO_2_ geosequestration and oil RF. This is complimented by rapid propagation of CO_2_ front at lower injected PV values and preferential flow across the fracture-matrix interface.

A detailed quantitative comparison between a probabilistic and a deterministic ML model has been carried out in the present study using the CFD data on CO_2_ saturation. Although both the models show close agreement with the actual S_CO2_ data, the predictive performance of GPR model has been shown to be superior of the XGBoost model. Scatter and residual plots, along with histograms of absolute error, R^2^ and RMSE show that GPR predicts CO_2_ saturation more accurately than XGBoost, while prediction bias has been noticed in XGBoost. Thus, it is concluded that GPR is the preferred model for characterising CO_2_-EOR in naturally fractured reservoirs. The hybrid approach developed in this study clearly demonstrate the feasibility of employing data-driven surrogate models for optimising CO_2_-EOR operations and enhancing CO_2_ geosequestration in geological formation.

## Data Availability

The authors declare that the data supporting the findings of this study are available within the paper. Moreover, codes developed for machine learning models are available from the corresponding author on reasonable request.
